# Bushen Yijing Fang Reduces Fall Risk in Late Postmenopausal Women with Osteopenia: A Randomized Double-blind and Placebo-controlled Trial

**DOI:** 10.1038/s41598-018-38335-3

**Published:** 2019-02-14

**Authors:** Yuxin Zheng, Xuezong Wang, Zong-Kang Zhang, Baosheng Guo, Lei Dang, Bing He, Chi Zhang, Jiwei Zhou, Wanzhong Shi, Yongfang Zhao, Hongsheng Zhan, Yu Xu, Chao Liang, Jin Liu, Daogang Guan, Luyao Wang, Xiaohao Wu, Jie Li, Zhenjian Zhuo, Zhixiu Lin, Hong Qiu, Lidan Zhong, Zhaoxiang Bian, Yinyu Shi, Bao-Ting Zhang, Ge Zhang, Aiping Lu

**Affiliations:** 1 Department of Orthopedics and Traumatology, Shuguang Hospital Affiliated to Shanghai University of Traditional Chinese Medicine, Shanghai, China; 2 School of Chinese Medicine, Faculty of Medicine, The Chinese University of Hong Kong, Hong Kong SAR, China; 3 Institute for Advancing Translational Medicine in Bone & Joint Diseases, School of Chinese Medicine, Hong Kong Baptist University, Hong Kong SAR, China; 4 Institute of Integrated Bioinformedicine and Translational Science, School of Chinese Medicine, Hong Kong Baptist University, Hong Kong SAR, China; 5 School of Basic Medicine, Shanghai University of Traditional Chinese Medicine, Shanghai, China; 6 Preparation Center of Traditional Chinese Medicine, Shuguang Hospital Affiliated to Shanghai University of Traditional Chinese Medicine, Shanghai, China; 7 Institute of Orthopaedics and Traumatology, Shanghai Academy of Traditional Chinese Medicine, Shanghai, China; 8 School of Public Health, Li Ka Shing Faculty of Medicine, The University of Hong Kong, Hong Kong SAR, China

## Abstract

Falls in late postmenopausal women with osteopenia usually cause fractures with severe consequences. This 36-month randomized, double-blind and placebo-controlled trial with a 10-year observational follow-up study aimed to investigate the long-term effect of herbal formula Bushen Yijing Fang (BSYJF) on fall risk in the late postmenopausal women with osteopenia. 140 late postmenopausal women (Femoral neck T-score, −2.5~−2 SD) were recruited and randomized to orally receive calcium carbonate 300 mg daily with either BSYJF or placebo for 36 months. The effect was further investigated for another 10-year follow-up. During the 36-month administration, there were 12 falls in BSYJF group and 28 falls in placebo group, respectively, indicating 64% lower risk of falls (RR 0.36 [95% CI, 0.18 to 0.71]; *P* = 0.004) in BSYJF group. During the 10-year follow-up, 36% lower fall risk (RR 0.64 [95% CI, 0.46 to 0.89]; *P* = 0.009) was observed in BSYJF group. No significant difference was found in safety profile between two groups. Thirty-six-month administration of BSYJF reduced fall risk with an increase in bone mass, and its latent effect on fall risk was continually observed in the 10-year follow-up in late postmenopausal women with osteopenia. This clinical trial was registered at Chinese clinical trial registry (ChiCTR-IOR-16008942).

## Introduction

Falls commonly happen in late postmenopausal women^1^. Fall-related fractures in late postmenopausal women, *e.g*. hip fracture, usually cause disability and even mortality^2,3^, which could be induced by a combination of bone-dependent factors and falls^3–5^. Bone mineral density (BMD) is commonly used to diagnose osteoporosis (T score ≤ −2.5) and to predict individual fracture risk^6^. However, many fractures occur in postmenopausal women who have non-osteoporotic BMD values (T score > −2.5)^7^. Therefore, the bone-independent risk factor, such as the tendency to fall, needs to be taken into consideration to reduce fall-related fractures in late postmenopausal women with osteopenia. Both decreased muscle mass and impaired functional mobility (muscular and neurological) are two important risk contributors to falls^8^. Thus, the two contributors should be targeted to reduce fall risk for preventing fall-related fracture in late postmenopausal women with osteopenia.

To date, there is still no effective pharmacological agent to prevent falls in clinical. Many clinical trials have been performed to study the effects of Vitamin D on fracture and falls^9,10^. Although Vitamin D supplements show beneficial effects on muscle function^5,11^. However, a recently updated meta-analysis concludes that vitamin D supplements do not prevent fracture and falls^12^. Therefore, it is desirable to develop a therapeutic agent which has potential to reduce falls.

The Chinese herbal formula “Bushen Yijing Fang” (BSYJF), approved by China Food and Drug Administration (CFDA No. Z20090656) and commercially available as a patent drug, has been prescribed as “bone-invigorating” and “muscle-strengthening” drug by Practitioners to treat musculoskeletal disorders in China^13^. Our experimental data in aged ovariectomized (OVX) rats showed that BSYJF could attenuate the decrease of muscle fiber cross-sectional area (CSA), the twitch force of extensor digitorum longus (EDL) (Appendix Fig. 1) and elevate the mRNA of myosin heavy chain that responsible for performing sustained and tonic contractile activities^14–16^. Furthermore, BSYJF showed beneficial effect on neurological function in aging rats^17^. The evidence implied the potential of BSYJF to both maintain the muscle mass and improve the functional mobility muscularly and neurologically. Besides of the animal data, the short-term clinical observation also demonstrated that BSYJF could potentially increase bone mass in both osteoporotic rodents and postmenopausal women^18–22^.

In this study, we performed a 36-month randomized, double-blind and placebo-controlled trial in late postmenopausal women with osteopenia to investigate efficacy of BSYJF on fall risk and long-term safety in the 36-month administration. Further, the latent effect on fall risk was also evaluated in a 10-year observational follow-up after completing the 36-month trial. Moreover, we explored and validated the underlying mechanism of BSYJF by bioinformatics analysis.

## Methods

### Study design

This study was a 36-month randomized, double-blind and placebo-controlled trial with a 10-year observational follow-up. As the trial registration regulation wasn’t implemented in China when this trial commenced in 2000, this study was retrospectively registered in Chinese clinical trial registry (ChiCTR-IOR-16008942; Date of Registration: 30/07/2016). The original study design didn’t include a 10-year observational follow-up. To further investigate the latent effect of BSYJF on fall risk in late post-menopausal women, we conducted a 10-year observational follow-up after completing the 36-month trial. All experimental protocols were approved by the local ethics committee of Shanghai Academy of Traditional Chinese Medicine and all methods were performed in accordance with the relevant guidelines and regulations (Approval No. SZYGSLL-1999-002).

### Participants and setting

This trial was conducted in two sites in Shanghai (Shuguang Hospital Affiliated to Shanghai University of Traditional Chinese Medicine & Institute of Orthopedics and Traumatology in Shanghai Academy of Traditional Chinese Medicine). All the participants were recruited via advertisement and provided written informed consent. Community-dwelling women (postmenopausal ≥10 years, aged 55~69 years) were invited via medical records and outreach materials. The women who had femoral neck T-score between −2.5~−2 SD for 12 months were included. The subjects were excluded if they (1) were diagnosed with neurological or musculoskeletal disorder, or chronic diseases, (2) took estrogen, calcitonin, fluoride, bisphosphonates, or adrenocortical hormone within one year, (3) took ≥ 4 prescription medications, (4) had postural hypotension: drop in systolic blood pressure ≥ 20 mmHg or to < 90 mmHg on standing, (5) had environmental hazards for falls or tripping, (6) had impairment in gait, (7) had impairment in transfer skills or balance, (8) had impairment in leg or arm muscle strength or range of motion (hip, ankle, knee, shoulder, hand, or elbow), (9) had alanine aminotransferase (ALT) or aspartate aminotransferase (AST) levels greater than 50% of upper normal limit, (10) had serum creatinine levels greater than 133 μmmol/l or 1.5 mg/dl (Appendix Table 1). All the included participants were provided a detailed written description about the study and an informed consent form before agreeing to participate and confirming eligibility. To protect the subjects who were randomized to placebo during the initial 36-month treatment phase, those who experienced a dramatic bone loss or fragility fracture were also excluded during 10-year observational follow-up.

### Allocation, randomization and blinding

The participants were randomly assigned to either BSYJF or placebo group by using the randomization-number table with a 1:1 ratio. Briefly, randomization numbers were generated by an independent statistician from the department of statistics, Shanghai University of Traditional Chinese Medicine. This statistician was not involved in following data collection or analysis. The codes were concealed in sealed, opaque envelopes with date and signature labels. All the participants, investigators and coordinators were blinded to the treatment assignment. The blinding codes were given to a pharmacist to keep and could not be broken unless in emergency situations during the study. The treatment arrangements were made by the pharmacist in each center, who was blinded to the participants’ characteristics and not involved in the number generation and recruitment process.

### Intervention and compliance

The participants were orally given calcium carbonate 300 mg daily with either BSYJF or placebo capsules (0.55 g/capsule, 3 capsules/time, 3 times/day) for 36 months continuously. The main ingredients in placebo were starch supplemented with food colorants and flavoring agents to mimic BSYJF capsules. Placebo capsules were identical to BSYJF capsules in size, weight, appearance, color and flavor.

The compliance was calculated at capsule collecting every three months (actual capsules consumption/required capsules consumption × 100%). The participants were considered evaluable if they were within 80% and 120% of compliance. All participants were arranged to join the Health Club and gathered once a month to facilitate the interaction between the participants and doctors.

### Quality control for BSYJF capsules

The BSYJF is composed of 7 herbs, *Herba Epimedii* (Yinyanghuo [in Chinese], stem leaves of *Epimedium brevicornum Maxim*.), *Radix Polygoni Multiflori* (Heshouwu, root tuber of Polygonum multiflorum Thunb.), *Herba Cistanches* (Roucongrong, stem of *Cistanche deserticola* Y. C. Ma), *Radix Astragali* (Huangqi, root of *Astragalus membranaceus*), *Rhizoma Drynariae* (Gusuibu, rhizome of *Drynaria fortune* J. Sm.), *Herba Dendrobii* (Shihu, stem of *Dendrobium nobile* Lindl.) and *Flos Chrysanthemi* (Juhua, flower head of *Chrysanthemum morifolium* Ramat.), with a weight ratio in 10:6:6:10:6:6:6. The manufacturing processes of BSYJF capsules were strictly complied with Good Manufacturing Practice (GMP) standards and Chinese Pharmacopoeia. In addition, the stability and consistency of the components in different batches of BSYJF capsule used in this study were also confirmed by chromatographic fingerprint (Appendix Table 2). The BSYJF was recorded in National Drug Standard of CFDA (No. YBZ10052009) and available from Xia’men Chinese Medicine Factory Co., LTD (Trade name, Qi-Gu Capsule).

### Endpoints

The primary endpoint was the number of falls at 36 months. Secondary endpoints were Timed Up and Go (TUG) test, lean mass of left thigh, femoral neck BMD, serum osteocalcin (OC), urine deoxypyridinoline (DPD), serum 25-OH-vitamin D (25(OH)D), 17 β-estradiol (E2) and endometrial thickness at 36 months. During the 36-month trial, the participants underwent the above assessment at baseline and 12, 24, 36 months.

Two independent orthopedic surgeons (Y.Z. and G.Z.) who were blinded to the characteristics of the participants performed the assessments.

#### Number of falls, fallers and fall-related hip fractures

A fall was defined as any event that led to an unplanned, unexpected contact with a supporting surface^23^. Falls resulting from pathologic fracture, major trauma or facial trauma, and/or unavoidable hazards such as transient ischemic attacks, chair collapsing or cerebrovascular accidents were excluded. A faller was defined as the participants sustained at least one fall or more. The participants were educated to record the information of new falls in the form immediately, including the date of fall and related-injury. The forms were collected at each clinical visit or monthly meeting in the health club for the 36-month trial and 10-year follow-up. The number of falls was accumulated at each time point^24^. The cumulative incidence of fallers was calculated as the total number of participants with first fall at each time point divided by the number of participants at risk^25^. The number of fall-related fractures was also recorded. A copy of radiograph confirming the fracture was obtained.

#### TUG test

The TUG test was designed to evaluate the functional mobility of the participants. The TUG test, consisting of both walking speed and chair rise components, was easy to complete by older adults^26^. Briefly, the participants were instructed to rise from a standard arm chair, walk 3 meters, turn around, walk back to the chair at the normal pace and sit down^23^. This test had high sensitivity (87%) and specialty (87%) to assess individual functional mobility^23^. Higher scores represented a poorer degree of functional mobility. The change of TUG score was normalized by the baseline.

#### Lean mass of left thigh and femoral neck BMD

All the densitometry operators in the two sites were specifically trained to ensure the standard procedure. The lean mass of left thigh and the BMD of left femoral neck were derived from the total body scan by Dual-energy X-ray Absorptiometry (DXA) (DPX-L; Lunar, Madison, WI, USA)^27^. The same DXA apparatus was used for the same participant over the entire period. Monitoring of DXA scanner drift was achieved by the regular scanning of a calibration phantom. The precision of a measurement was 1.86% for femoral neck and 0.74% for lean mass of left thigh. The changes of the femoral neck BMD and lean mass of left thigh were normalized by their corresponding baseline, respectively.

#### OC and urine DPD

The OC and urine DPD were bone turnover markers for predicting bone formation and bone resorption, respectively. The OC was assessed by a commercial ELISA Kit (Quidel, San Diego, CA, USA). Intraassay and interassay coefficient of variations (CVs) were 5.2% and 7.5% for serum OC, respectively. Urine DPD was quantified by a commercial ELISA Kit (Ostex International, Seattle, WA, USA). Intraassay and interassay CVs were 5.9% and 6.5% for urine DPD, respectively.

#### Serum 25(OH)D and E2

The serum 25(OH)D and E2 were also important indices of mineral metabolism. 25(OH)D concentrations were measured by a commercial radioreceptor assay (Amersham, Bucks, UK). Normal values for this age group were 4.3–40.5 ng/ml (within assay variation was 4–6%). E2 was assessed by a commercial RIA kit (Jiu Ding, Tianjing, China). Intraassay and interassay CVs were 4.0% and 5.0%, respectively.

#### Endometrial thickness

Endometrial thickness was measured at the thickest portion of the endometrium by transvaginal ultrasound (Siemens Medical Solutions Systems, Issaquah, WA, USA) and both endometrial layers were included. The sensitivity of detecting endometrial abnormalities was 92% when an endometrial thickness of 5 mm was used as the upper limit of the normal value^28^.

Fasting blood and urine samples were collected at each time point. The serum was separated from the blood corpuscles by centrifugation and stored at −70 °C until the assessment. All urine samples were analyzed for their creatinine content, and excretion of DPD was corrected for creatinine excretion. The changes of the OC and DPD were normalized by their corresponding baseline, respectively.

For safety, biochemical examination (complete blood count, renal function, and liver function), blood pressure and electrocardiogram, and observation of abnormal symptoms/signs based on standardized patient report forms were performed at baseline and 12, 24, 36 months. During each clinical visit, abnormalities were evaluated and documented, and follow-up medical care was provided as needed. Potential adverse events were monitored for each subject.

The participants, who successfully entered the treatment allocation but failed to complete the whole period, were considered as dropout cases. The reasons for dropout were recorded, and the last data of these participants were also included in the data analysis. The subjects could voluntarily withdraw at any time during the trial. The trial could be terminated if serious adverse events, protocol deviation or loss to follow-up occurred and/or BSYJF was found to have no clinical value after long-term administration.

During the entire trial, the subjects were advised to follow the guideline for daily dietary intake and physical activities, such as intaking high-protein diet, avoiding alcohol abuse and smoking, and keeping active in daily physical activities. The questionnaire for daily dietary intake and physical activities were recorded at baseline and 12, 24, 36 months during 36-month clinical trial. Calcium and vitamin D supplement on their own were prohibited in 36-month clinical trial.

### Ten-year follow-up

For the extension treatment-free follow-up phase, the participants were followed-up at the 3rd, 6th and 10th year to assess the latent effects of BSYJF on falls and fall-related hip fractures in late postmenopausal women. The number of falls, fallers and fall-related hip fractures were recorded and the serum 25(OH)D levels were measured at the 3rd, 6th and 10th year. The information of falls, fracture, daily dietary intake and physical activities were collected by phone call or health club gathering once a month.

### Statistical analysis

Assuming the fall incidence among community-dwelling elderly women was 19% in placebo group^29^, we hypothesized that BSYJF would reduce 50% in fall risk annually^30^. A sample size of 110 (55 per group) was required for 90% power at one-sided 5% significance level. Assuming the dropout of 20%, 70 participants for each group were adopted.

An intention-to-treat approach was used for all the analyses. The number of falls was presented as risk ratio (RR), determined by the Negative binomial with log link in generalized estimating equations (GEEs) with an unstructured covariance matrix. The rate of fallers at 36 months was presented as hazard ratio (HR), determined by a Cox regression analysis. The rate of fallers at 10-year follow-up, as well as the number of fall-related hip fractures, were presented as odds ratio (OR), determined by the Binary logistic regression with Hosmer-Lemeshow method. The Kaplan–Meier analysis was also performed to demonstrate differences in the time to the first fall between two groups. Censoring time was defined as the time from randomization until the last follow-up. A sensitivity analysis was performed by Negative binomial with log link in GEEs.

The continuous outcomes at different time points were analyzed by Linear model in GEEs. The differences between groups were calculated by analysis of covariance model (ANCOVA) or Wilcoxon rank-sum test. The difference within-group was analyzed by *t*-test. A sensitivity analysis was performed by Linear model in GEEs.

The age, time since menopause, body mass index (BMI) and history of fall at baseline were used as covariates. The analysis for adverse events was performed by chi-square test. All variables were presented as mean (SD) or median (IQR), and provided with 95% confidence interval (CI) or percentage. All tests were used two-sided and set at the 5% level using IBM SPSS 20.0.

## Results

### Baseline characteristics

**Figure 1:**
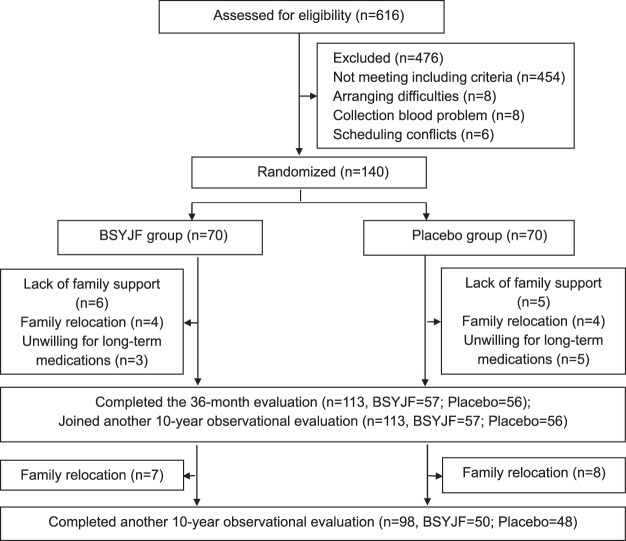
Flowchart of participants through the whole study. Note: BSYJF = Bushen Yijing Fang.

From 2000 to 2001, 616 subjects were recruited for screening and 140 participants were eligible (Fig. 1). The participants were randomly assigned to two groups. The number of subject recruited in each season in two groups was balanced. The demographic and key characteristics at baseline were well-balanced between two groups (Table 1).

**Table 1:** Baseline characteristics of the participants.

Variable	BSYJF (n = 70)	Placebo (n = 70)
**Demographics**		
Mean age (SD), *y*	63.91 (2.86)	63.90 (2.81)
Mean height (SD), *cm*	1.56 (0.05)	1.55 (0.05)
Mean weight (SD), *kg*	52.19 (4.41)	52.84 (3.92)
Mean BMI (SD), *kg/m*^2^	21.56 (1.51)	21.96 (1.46)
Mean years since menopause (SD), *y*	14.91 (2.41)	14.81 (2.47)
Fallers (%), *n*	2 (3%)	3 (4%)
**Disease conditions**		
Mean 25-OH-vitamin D (SD), *ng/mL*	17.84 (4.19)	17.87 (4.63)
Mean femoral neck BMD (SD), *g/cm*^2^	0.663 (0.029)	0.671 (0.018)
Median T-score of femoral neck (IQR)	−2.20 (0.20)	−2.20 (0.20)
Mean lean mass of left leg (SD), *kg*	5.47 (0.55)	5.50 (0.44)
Mean TUG test (SD), *s*	7.60 (0.48)	7.66 (0.48)
Mean deoxypyridinoline (SD), *nmol/mmol*	7.61 (1.21)	7.68 (1.12)
Mean osteocalcin (SD), *μg/L*	11.89 (2.33)	11.68 (2.16)
Mean estradiol (SD), *pmol/L*	36.36 (5.14)	35.09 (4.58)
Mean endometrial thickness (SD), *mm*	1.87 (0.31)	1.79 (0.28)

Note: BMI = body mass index; BMD = bone mineral density; TUG = Timed Up and Go, higher scores indicate more severe disease status; BSYJF = Bushen Yijing Fang.

### Dropout and compliance

For the 36-month trial, twenty-seven participants withdrew from the trial: 13/70 in BSYJF and 14/70 in placebo. The reasons were 1) lack of family support (6 [46%] in BSYJF and 5 [36%] in placebo); 2) family relocation (4 [31%] in BSYJF and 4 [28%] in placebo); 3) Unwilling for long-term medications (3 [23%] in BSYJF and 5 [36%] in placebo) (Fig. 1). The compliance of BSYJF/placebo capsules administration was 91.61% (SD 0.51%) and 91.55% (SD 0.54%), respectively (Appendix Table 3). For the 10-year observational follow-up, 15 participants were lost to follow-up: 7/57 in BSYJF and 8/56 in placebo The reason was family relocation (Fig. 1).

### Endpoints for 36-month trial

**Figure 2:**
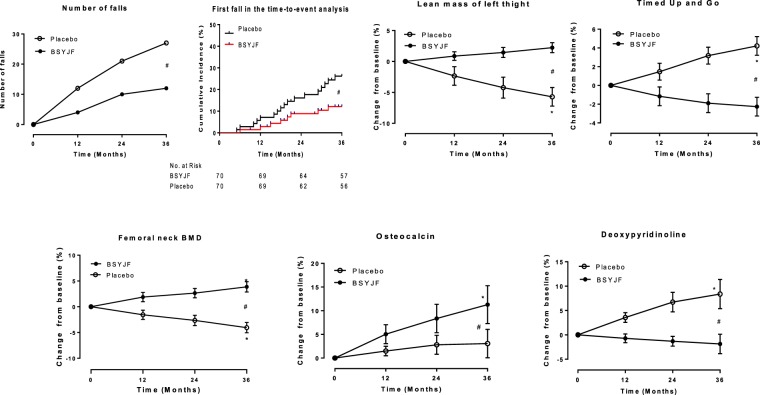
The change patterns of fall-related (upper panel) and bone-dependent (lower panel) fracture risk factors in late postmenopausal women during 36-month clinical trial. The fall-related fracture risk factors include the number of falls (upper left), the first fall in the time-to-event analysis (upper middle-left), lean mass (upper middle-right) and Timed Up and Go (TUG) score (upper right). The bone-dependent fracture risk factors include femoral neck BMD (lower left), serum osteocalcin levels (lower middle) and urine deoxypyridinoline levels (lower right). The Kaplan–Meier analysis was performed to demonstrate differences in the time to the first fall between two groups. Negative binomial model with log link was used to analyze the number of falls. The Linear model in generalized estimating equations (GEEs) analysis was used to analyze the change patterns over time between two groups. Error bars indicate pointwise 95% confidence intervals for the value of lean mass of left tight, and interquartile ranges for the values of formal neck BMD, TUG, and the levels of osteocalcin and deoxypyridinoline. Note: *P < 0.05 for within-group comparisons in BSYJF group or placebo group in percentage change from baseline. ^#^*P* < 0.05 for between-group difference at 36 months. BMD = bone mineral density, BSYJF = Bushen Yijing Fang; TUG = Timed Up and Go.

For the number of falls, there were 12 falls in BSYJF and 28 falls in placebo at 36 months, respectively. The fall risk was 64% lower in BSYJF than that in placebo (RR 0.36 [95% CI, 0.18 to 0.71]; *P* = 0.004). For the number of fallers, there were 8 fallers in BSYJF and 17 fallers in placebo at 36 months, respectively. The rate of fallers in BSYJF was 60% lower compared with that in placebo (HR 0.40 [95% CI, 0.17 to 0.95]; *P* = 0.038). For the number of fall-related hip fractures, there was 1 case in each group at 36 months, respectively (Fig. 2 and Table 2).

**Table 2:** The endpoints between BSYJF group and placebo group during 36-month intervention.

Variable	36-Month Intervention			
	BSYJF (n = 70)	Placebo (n = 70)	RR/HR/OR (95% CI)	*P* value
Falls, RR^†^	12	28	0.36 (0.18–0.71)	0.004
Fallers, HR^‡^	8	17	0.40 (0.17–0.95)	0.038
One fall	5	10	N/A	N/A
≥2 falls	3	7	N/A	N/A
Hip fractures, OR^§^	1	1	0.89 (0.50–15.84)	0.93
Mean TUG test (SD), s*	7.48 (0.41)	8.05 (0.40)	N/A	<0.001
Mean Lean mass of left thigh (SD), kg*	5.59 (0.15)	5.19 (0.12)	N/A	<0.001
Mean femoral neck BMD (SD), *g/cm*^2*^	0.698 (0.018)	0.646 (0.014)	N/A	<0.001
Mean osteocalcin (SD), *μg/L* *	13.59 (2.16)	11.71 (1.80)	N/A	0.005
Mean deoxypyridinoline (SD), *nmol/mmol*	7.57 (0.88)	8.49 (0.96)	N/A	<0.001
Mean 25-OH-vitamin D (SD), ng/mL*	15.76 (1.06)	15.23 (1.16)	N/A	>0.05
Mean estradiol (SD), pmol/L*	36.35 (3.79)	33.73 (3.49)	N/A	>0.05
Mean endometrial thickness (SD), mm*	1.79 (0.22)	1.67 (0.21)	N/A	>0.05

Note: BSYJF = Bushen Yijing Fang; N/A = not applicable.

^†^Negative binomial model with log link was used to analyze the number of falls with RR (95% CI).

^‡^Cox regression analysis was performed for fallers at 36-month intervention with HR (95% CI).

^§^Binary logistic regression with Hosmer-Lemeshow method was used to analyze fall-related hip fractures at 36-month intervention.

*Values were analyzed by analysis of covariance model (ANCOVA) at 36 months of trial.

The age, time since menopause, body mass index (BMI) and history of falls at baseline values were used as covariates.

There were significant differences between the BSYJF and placebo in the TUG score, lean mass of left thigh, femoral neck BMD and biochemical markers (OC and urine DPD) at 36 months, respectively (*P* ≤ 0.001 for interaction between time and group). Within each group, compared to the baseline, the TUG score in placebo significantly increased at 36 months (4.22%, *P* < 0.001), whereas BSYJF maintained TUG score (−2.26%, *P* = 0.071). The lean mass in placebo significantly decreased at 36 months (−5.71%, *P* < 0.001), whereas BSYJF maintained the lean mass (2.05%, *P* = 0.24). The femoral neck BMD in BSYJF was increased from baseline (3.86%, *P* < 0.001), whereas that in placebo was significantly decreased (−4.05%, *P* < 0.001). The OC levels did not change in placebo at 36 months (3.06%, *P* = 0.36), whereas BSYJF significantly increased OC at 36 months (11.28%, *P* = 0.001). The urine DPD levels increased in placebo at 36 months (8.38%, *P* = 0.001), whereas BSYJF slightly decreased DPD level at 36 months (−1.86%, *P* = 0.65) (Fig. 2, Table 2 and Appendix Table 4). There was no significant difference in the change patterns of 25(OH)D (*P* = 0.137), serum E2 levels (*P* = 0.53) and endometrial thickness (*P* = 0.27) between two groups during 36-month intervention, respectively (Table 2 and Appendix Table 5).

### Endpoints for 10-year extension follow-up

At 10-year extension follow-up phase, there were 81 falls in BSYJF and 123 falls in placebo, respectively. The fall risk was 36% lower in BSYJF than that in placebo (RR 0.64 [95% CI, 0.46 to 0.89]; *P* = 0.009). For the number of fallers, there were 31 fallers in BSYJF and 33 fallers in placebo at 10-year follow-up, respectively. The rate of fallers in BSYJF was 25% lower compared with that in placebo (OR 0.75 [95% CI, 0.32 to 1.79]; *P* = 0.52). For the number of fall-related hip fractures, there were 1 case in BSYJF and 3 cases in placebo, respectively. There was 77% reduction in hip fracture rate in BSYJF during the 10-year extension follow-up (OR 0.23 [95% CI, 0.02 to 2.98]; *P* = 0.26) (Table 3).

**Table 3:** The endpoints between BSYJF group and placebo group during 10-year extension follow-up.

Variable	10-Year Extension Follow-up			
	BSYJF (n = 57)	Placebo (n = 56)	RR/OR (95% CI)	*P* value
Falls, RR^†^	81	123	0.64 (0.46–0.89)	0.009
Fallers, OR^§^	31	33	0.75 (0.32–1.79)	0.52
One fall	3	2	N/A	N/A
≥2 falls	28	31	N/A	N/A
Hip fractures, OR^§^	1	3	0.23 (0.02–2.98)	0.26
Mean 25(OH)D (SD), ng/mL*	12.63 (0.96)	12.89 (1.11)	N/A	>0.05

Note: BSYJF = Bushen Yijing Fang; N/A = not applicable.

^†^Negative binomial model with log link was used to analyze the number of falls with RR (95% CI).

^§^Binary logistic regression with Hosmer-Lemeshow method was used to analyze the fallers and fall-related hip fractures at 10-year extension follow-up with OR (95% CI).

*Values were analyzed by analysis of covariance model (ANCOVA) at 10^th^ year of follow-up.

The age, time since menopause, body mass index (BMI) and history of falls at baseline values were used as covariates.

There was no significant difference in the change patterns of 25(OH)D between two groups during 10-year follow-up (*P* = 0.06) (Table 3 and Appendix Table 6). The serum 25(OH)D levels in both groups slightly decreased during the whole study.

### Daily dietary intake and physical activities

Both the time of daily physical activity and the calcium/vitamin D intake between two groups was similar during 36-month clinical trial and 10-year extension follow-up.

### Adverse events

Related adverse events were summarized in Table 4. In those tolerable adverse events, 9 participants were reported in BSYJF group and 8 in placebo group (13% vs. 11%; RR, 1.13 [95% CI, 0.46 to 2.75]; *P* = 0.80). Gastrointestinal complaints, such as stomach discomfort, constipation, and nausea/vomiting, were the most common complaints (5 in BSYJF and 4 in placebo).

**Table 4:** Related adverse events during the 36-month study period.

Adverse events	BSYJF (n = 70)	Placebo (n = 70)
Gastrointestinal complaints	5	4
Liver enzyme abnormal	2	1
Eczema	1	2
Hypertension	1	0
Breast uncomfort	0	1
Total adverse events (%)	9 (13%)	8 (11%)

Note: BSYJF = Bushen Yijing Fang.

The percent of adverse events = the number of adverse events/the number of participants at risk.

Minimally elevated liver enzyme levels were found in 2 women in BSYJF and 1 woman in placebo. The elevated liver enzyme levels returned to the normal range by the next test in all the 3 participants.

### Sensitivity analysis

Sensitivity analysis showed no change in the number of falls at 36 months (RR 0.41[95% CI, 0.17 to 0.99]; *P* = 0.048) and other outcomes (Appendix Table 7).

## Discussion

The study is the first randomized, double-blind and placebo-controlled trial with a 10-year observational follow-up to investigate the long-term effect of Chinese herb formula on fall risk in the late postmenopausal women with osteopenia.

With the increasing age, fragility fractures in the postmenopausal women is an ongoing concern for orthopaedic surgeons. The most common sites for fragility fracture are the vertebrae, hip (proximal femur) and wrist (distal forearm). Unlike fractures at other two sites, vertebral fractures are the most common fragility fractures caused by osteoporosis, only a minority of vertebral fractures result from a fall^31^. Conversely, most fall-related fractures are nonvertebral ones, and the site and type of fracture are dependent on the direction of the fall. Falling sideways or straight down is more likely to result in a hip fracture^32,33^, while falling backwards or obliquely forward is more likely to cause a distal forearm fracture^34^. However, distal forearm fractures do not tend to occur in frail individuals, since the elderly tend to move slowly and are unable to put out their hand to break a fall, in turn, decrease their risk for distal forearm fracture^35,36^. Therefore, hip fracture is the most representative as the fall-related fragility fractures. In the current trial, totally 7 hip fractures were reported.

BSYJF reduced fall risk, evidenced by the lowered fall number in BSYJF group during 36-month trial. Surprisingly, its latent effect on fall risk was still observed in 10-year follow-up in late postmenopausal women with osteopenia. Muscle mass and functional mobility were recognized as significant predictors for falls^37^. The lowered number of falls could be partially explained by the maintained thigh lean mass and TUG score in those women treated with BSYJF. The beneficial effect of BSYJF on muscle was similar with our animal data. BSYJF also modified bone-dependent risk factors, evidenced by the increased femoral neck BMD in BSYJF group at 36 months. It might be explained by the promoted bone formation (promoted increase in serum OC level) and attenuated bone resorption (prevented increase in urine DPD level) in the late postmenopausal women treated with BSYJF.

It has been reported that other risk factors (*e.g*., impairment of gait or balance, neurological disorders and environment hazards) also contribute to falls^38^. In this clinical study, these potential risk factors were excluded at baseline before recruitment. There is seasonal difference in falls and fractures among the elderly, more falls and fractures with more severe outcomes occur in winter^39^. In our study, the number of subject recruited in each season in two groups was balanced, which the influence of season factor could be considered minimized. Recently, daily physical activities and daily dietary intake, especially calcium and vitamin D intake, have been demonstrated to influence fall risk^40^. During the 36-month clinical trial and 10-year follow-up, the daily dietary intake and physical activities between two groups were similar. Serum 25(OH)D also showed similar level between two groups. After excluding the above potential risk factors, the difference in fall risk between the two groups could be associated with BSYJF.

According to the bioinformatics analysis, seven herbs in BSYJF were classified into two categories based on their target genes in fall-related network, herbs target ≥ 3 genes (HT3G) or herbs target ≤ 2 genes (HT2G) (Supplement). HT3G was demonstrated to be the active component within BSYJF in regulating muscle mass and muscle strength, evidenced by the unimproved muscle property in OVX rats after HT2G treatment. However, HT2G within BSYJF was not expendable due to its small target gene number. Bone became fragile after treatment of BSYJF without HT2G. These evidence implied that the seven herbs within BSYJF should be used as a whole to modify fall-related fracture risk factors.

To date, the guidance for fracture prevention in postmenopausal osteopenic women mainly focuses on the bone-dependent risk factors, the recommended therapy is calcium plus vitamin D supplements^41^. Different interventions have shown some beneficial effect in clinical trials to prevent falls among the elderly, including exercises (e.g., Tai Chi^42^), environment modification^43^, medication^9^ or the combination interventions^44^. Although vitamin D supplements exert a small positive impact on lower limb muscle strength (standardized mean difference [SMD] of 0.19 kg force; 95% CI, 0.05 to 0.34; 19 trials), a significant heterogeneity in the meta-analysis is reported^45^ and no effect is found in either muscle mass (SMD of 0.058 kg; 6 trials)^45^ or TUG (SMD of 0.3 s; 95% CI, 0.1 s to 0.5 s; 5 trials)^46^. In addition, the updated meta-analyses show that vitamin D supplements have no effects on falls (RR 0.98 [95% CI, 0.94 to 1.02]; 23 trials with more than 30 000 participants)^47^. In current study, BSYJF treatment did not affect the serum 25(OH)D levels during the whole study, implying BSYJF exerts beneficial effects independent of vitamin D. Therefore, BSYJF may have a greater advantage than vitamin D supplement on modifying fall-related fracture risk factors in late postmenopausal women with osteopenia.

BSYJF was not associated with a detectable hyperplasia and estrogen-like effect on the uterus in this study, which was evidenced by no significant change in endometrial thickness and serum E2 in BSYJF-treated women. The findings implied the tissue selectivity of the BSYJF containing phytoestrogenic compounds. The results were consistent with one of our previous published clinical trial of epimedium, one of the major herbs in BSYJF^48^.

No severe adverse events occurred during treatment period. The number of gastrointestinal complaints in BSYJF was close to that in placebo, which was consistent with the findings in previous trial^49^ and another study of Chinese herbal formula (Xianlinggubao, XLGB)^50^. No significant difference in the complaints with minimally elevated liver enzyme levels between two groups was observed, although a meta-analysis suggested that phytoestrogens were with moderately increased risk of adverse gastrointestinal effects compared with placebo^51^. Consistently, no prominent adverse events of BSYJF were reported by CFDA (http://eng.cfda.gov.cn/; No. Z20090656).

Limitations of this research were noted as follows: (1) Given the fact that fall risk was multifactorial^40^, some fall related factors, such as lifestyles and cognitive abilities, were not taken into consideration in this study. (2) Lack of information concerning participants’ physical activity, co-morbidities and medications during the 10-year extension follow-up phase limited the cause–effect relationship between BSYJF and outcomes.

In conclusion, 36-month administration of BSYJF reduced fall risk with an increase in femoral neck bone mass, and its latent effect on fall risk was continually observed in 10-year follow-up in late postmenopausal women with osteopenia.

## Supplementary information

Appendices

Protocol

## Data Availability

All data generated or analysed during this study are included in this published article and its Supplementary Information.
