# Supplementary material for: Bushen Yijing Fang Reduces Fall Risk in Late Postmenopausal Women with Osteopenia: A Randomized Double-blind and Placebo-controlled Trial
Source: Sci Rep. 2019 Feb 14;9:2089. doi: 10.1038/s41598-018-38335-3 (PMC6375933; doi:10.1038/s41598-018-38335-3)
Supplement: Supplementary file 1 — Appendices [file 41598_2018_38335_MOESM1_ESM.pdf]

Bushen Yijing Fang Reduces Fall Risk in Late Postmenopausal Women with Osteopenia: A Randomized Double-blind and Placebo-controlled Trial

Yuxin Zheng[1,+], Xuezong Wang[1,+], Zong-Kang Zhang[2,+], Baosheng Guo[3,5,+], Lei Dang[3,+], Bing He[3,4], Chi Zhang[3], Jiwei Zhou[1], Wanzhong Shi[6], Yongfang Zhao[7], Hongsheng Zhan[7], Yu Xu[7], Chao Liang[3], Jin Liu[3], Daogang Guan[4], Luyao Wang[3], Xiaohao Wu[3], Jie Li[2], Zhenjian Zhuo[2], Zhixiu Lin[2], Hong Qiu[8], Lidan Zhong[3], Zhaoxiang Bian[3], Yinyu Shi[1], Bao-Ting Zhang[2,*], Ge Zhang[3,*], Aiping Lu[3,4,5,*],

[1]Department of Orthopedics and Traumatology, Shuguang Hospital Affiliated to Shanghai University of Traditional Chinese Medicine, Shanghai, China;

[2]School of Chinese Medicine, Faculty of Medicine, The Chinese University of Hong Kong, Hong Kong SAR, China;

[3]Institute for Advancing Translational Medicine in Bone & Joint Diseases, School of Chinese Medicine, Hong Kong Baptist University, Hong Kong SAR, China;

[4]Institute of Integrated Bioinformedicine and Translational Science, School of Chinese Medicine, Hong Kong Baptist University, Hong Kong SAR, China;

[5]School of Basic Medicine, Shanghai University of Traditional Chinese Medicine, Shanghai, China;

[6]Preparation Center of Traditional Chinese Medicine, Shuguang Hospital Affiliated to Shanghai University of Traditional Chinese Medicine, Shanghai, China;

[7]Institute of Orthopaedics and Traumatology, Shanghai Academy of Traditional Chinese Medicine, Shanghai, China;

[8]School of Public Health, Li Ka Shing Faculty of Medicine, The University of Hong Kong, Hong Kong SAR, China;

[+]These authors contributed equally to this study.

*Correspondence:

Bao-Ting Zhang, Tel: 852-3943-4285; Fax: 852-3942-0941; Email: zhangbaoting@cuhk.edu.hk

Ge Zhang, Tel: 852-3411-2457; Fax: 852-3411-2461; Email: zhangge@hkbu.edu.hk

Aiping Lu, Tel: 852-3411-2457; Fax: 852-3411-2461; E-mail: aipinglu@hkbu.edu.hk

Contents

Appendix 1 Methods and Results of animal studies: 1

Appendix Figure 1 Effect of BSYJF on skeletal muscle in OVX rat 2

Appendix Table 1 Inclusion and exclusion criteria 3

Appendix Table 2 Resemblance analysis of chemical composition in BSYJF from different batches of capsules. 4

Appendix Table 3 Compliance of the clinical trial 5

Appendix Table 4 Summary data for changes of bone- and fall-related variables after 36 months. 6

Appendix Table 5 Summary data for changes of 25(OH)D, Endometrial thickness and E2 after 36 months. 8

Appendix Table 6 Summary data for changes of 25(OH)D after 10-year observational follow-up. 9

Appendix Table 7 Sensitivity analyses for secondary endpoints. 10

Appendix 2 Methods and Results of Bioinformatics Analysis and *In Vivo* Validation 12

Methods 12

Results 13

Appendix Table 8 Identification of chemical constituents in BSYJF capsules using UPLC-Q-TOF MS in either negative or positive ion modes. 14

Appendix Table 9 Bone-dependent and fall-related functions of target genes of chemical constituents in BSYJF. 15

Appendix Figure 2 Bioinformatic analysis results. 17

Appendix Figure 3 Role of HT3G within BSYJF in regulating muscle mass and strength in OVX rat. 21

Appendix Figure 4 Role of HT2G within BSYJF in regulating bone mechanical property in OVX rat. 22

Appendix 1 Methods and Results of animal studies:

Methods

Study design: All protocols and experimental procedures were approved by the Animal Experimentation Ethics Committee of the Chinese University of Hong Kong. All experiments were performed in accordance with the relevant guidelines and regulations outlined in the Animal Experimentation Ethics Committee Guide for the Care and Use of Laboratory Animals, The Chinese University of Hong Kong. Twenty-four 6-month old female Sprague Dawley (SD) rats were recruited. Ovariectomy was performed to mimic postmenopause. Eight rats were sacrificed as baseline group (BL) at 6 months post-surgery. The rest rats were assigned into 2 groups as follows, PBS group (n=8): daily oral administration of 2 ml PBS for three months started from 6 months post-surgery; BSYJF group (n=8): daily oral administration of BSYJF powder in PBS (100mg/kg) for three months started from 6 months post-surgery. All the rats were sacrificed after 3-month intervention. The left extensor digitorum longus (EDL) was collected for *in vitro* muscle testing and the right EDL was collected for hematoxylin and eosin (H&E) staining to determine the cross-sectional area (CSA) of the muscle fiber.

Evaluation protocols:

***In vitro* muscle functional testing:** Dissected muscle with intact tendons was mounted in a specific chamber bathed with 95%O2/5%CO2-bubbled Krebs' solution (In Vitro Muscle Test System 1205, Aurora Scientific Inc., Aurora, ON, Canada). One end of the tendon was attached to a hook connected to the lever arm of a position feedback motor, and the other end was attached to a force transducer. The muscle was stimulated with 0.5 ms pulses of supramaximal intensity through two platinum plates that were parallel to the muscle. Peak twitch force at 1Hz and peak tetanic force at 100Hz for 400 ms were recorded with the ASI Dynamic Muscle Control Software (DMC v5.1 beta, Aurora Scientific Inc.).

Cryosectioning and H&E staining: The dissected soleus muscles were snap frozen in liquid nitrogen-cooled isopentane and then embedded in OCT medium. Serial cross-sections (6 μm thickness) were cut from the mid-belly of the muscles on a cryostat at -20°C for histological and immunohistochemical staining. H&E staining was performed on sections to examine the general morphology. The software ImageJ was used to calculate the muscle fiber CSA.

Three-point bending test: The three-point bending test was performed using the materials testing system (MTS, Eden Prairie, MN USA) following the established procedure. The femur was placed on its lateral surface on two rounded supporting bars that were 20 mm from each other. A preload of 1 N was applied by lowering the upper bar. A constant displacement rate of 6 mm/min was applied until bone break. Energy at ultimate force, determined by the area under the curve until ultimate force, was calculated.

Results:

The muscle fiber CSA of EDL from rats in PBS group was significantly decreased after the ovariectomy. BSYJF treatment attenuated the decline of muscle fiber CSA in aged OVX rats. In vitro muscle testing showed the twitch force of EDL in PBS group was dramatically decreased, while the twitch force of EDL in BSYJF group was significantly higher than that in PBS group. The time to peak (TTP) of EDL was unaltered (**Appendix Figure 1**).

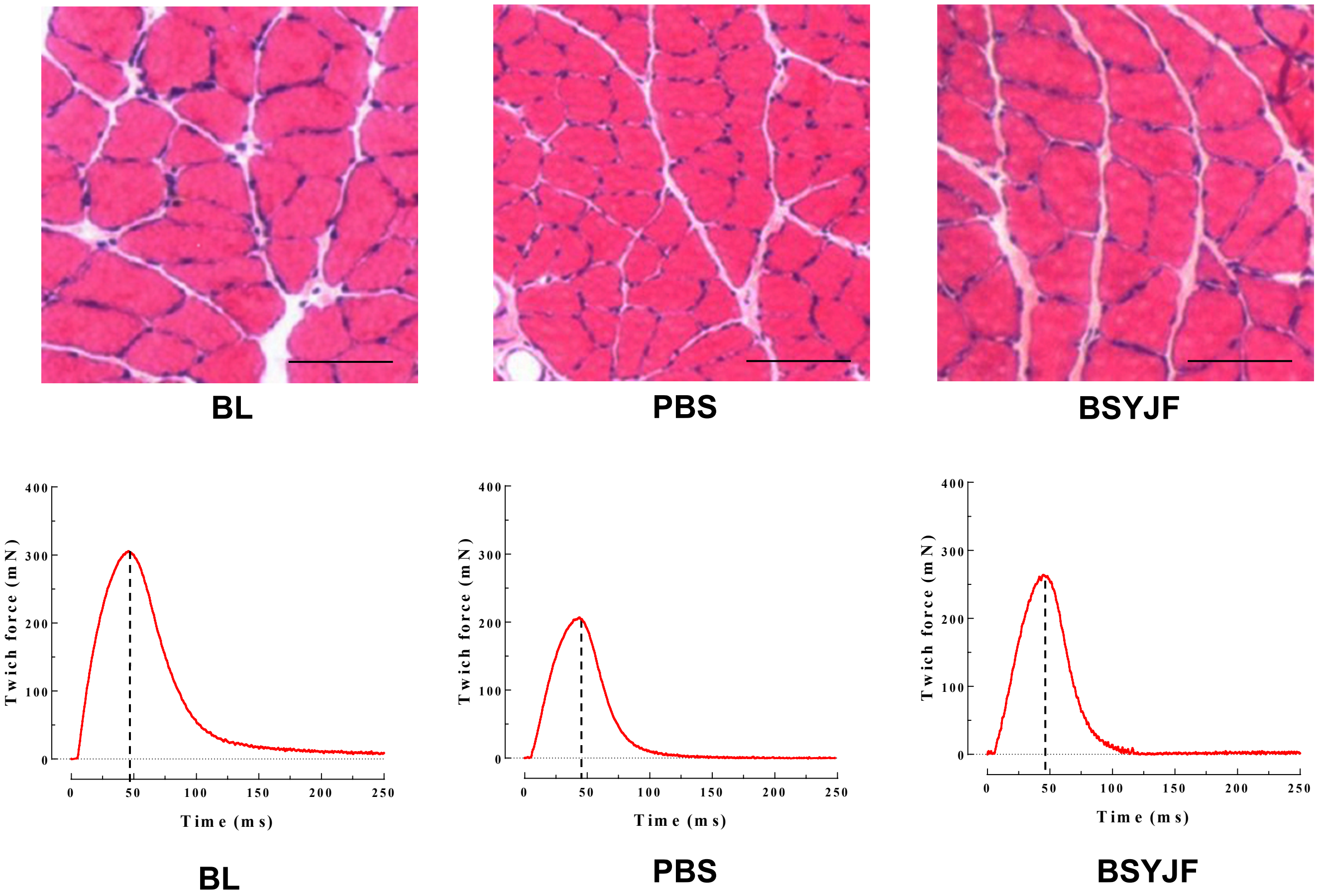

Appendix Figure 1 Effect of BSYJF on skeletal muscle in OVX rat. Representative H&E staining of muscle fiber in extensor digitorum longus (EDL) from indicated groups (bar=50μm) (top). Muscle twitch force and time to peak (TTP) of EDL from indicated groups (bottom). **Note:** BL= Baseline; BSYJF= Bushen Yijing Fang.

Appendix Table 1 Inclusion and exclusion criteria

Inclusion criteria	Exclusion criteria
• community-dwelling women;<br>• ≥10 years postmenopausal (aged 55~69 years) ;<br>• −2.5 SD ≤T-score ≤ −2 SD at femoral neck.	• diagnosed with neurological or musculoskeletal disorder;<br>• took estrogen, calcitonin, or bisphosphonates within one year;<br>• took ≥4 prescription medication;<br>• had postural hypotension;<br>• had environmental hazards for falls;<br>• had impairment in gait;<br>• had impairment in transfer skills or balance;<br>• had impairment in leg or arm muscle strength or range of motion;<br>• had ALT or AST levels >50% of upper normal limit;<br>• had serum creatinine levels >133μmmol/l or 1.5 mg/dl.

Appendix Table 2 Resemblance analysis of chemical composition in BSYJF from different batches of capsules.

Batch	Batch No.	S1	S2	S3	S4	S5	S6	S7	S8	S9	S10	Semblance
**S1**	160201	1	0.957	0.954	0.98	0.98	0.951	0.952	0.955	0.962	0.951	0.976
**S2**	150302	0.957	1	0.982	0.982	0.983	0.99	0.988	0.992	0.988	0.942	0.994
**S3**	150501	0.954	0.982	1	0.967	0.97	0.974	0.968	0.971	0.965	0.901	0.978
**S4**	151101	0.98	0.982	0.967	1	0.997	0.978	0.976	0.98	0.978	0.944	0.991
**S5**	151103	0.98	0.983	0.97	0.997	1	0.981	0.982	0.984	0.985	0.944	0.994
**S6**	160502	0.951	0.99	0.974	0.978	0.981	1	0.998	0.999	0.993	0.926	0.993
**S7**	160601	0.952	0.988	0.968	0.976	0.982	0.998	1	0.999	0.998	0.935	0.994
**S8**	160503	0.955	0.992	0.971	0.98	0.984	0.999	0.999	1	0.997	0.938	0.995
**S9**	160501	0.962	0.988	0.965	0.978	0.985	0.993	0.998	0.997	1	0.951	0.995
**S10**	150201	0.951	0.942	0.901	0.944	0.944	0.926	0.935	0.938	0.951	1	0.956
**Semblance**	-	0.976	0.994	0.978	0.991	0.994	0.993	0.994	0.995	0.995	0.956	1

Note: BSYJF= Bushen Yijing Fang

Appendix Table 3 Compliance of the clinical trial

	BSYJF (%)	Placebo (%)
**BSYJF capsules**	91.61±0.51	91.55±0.54
**Calcium tablets**	92.02±0.48	91.80±0.61

Note: BSYJF= Bushen Yijing Fang.

Appendix Table 4 Summary data for changes of bone- and fall-related variables after 36 months.

Variable	BSYJF(n=70)	Placebo(n=70)	Between-Group Difference		Time-Group Interaction
			BSYJF vs. Placebo	*P* value**	*P* value*
Lean mass of left thigh, *kg*†					<0.001
Month 12	5.54(5.40 to 5.67)	5.37(5.27 to 5.48)	0.16(0.01 to 0.33)	0.032§	N/A
Month 24	5.56(5.43 to 5.70)	5.29(5.18 to 5.40)	0.27(0.10 to 0.45)	0.001§	N/A
Month 36	5.59(5.43 to 5.74)	5.19(5.07 to 5.31)	0.39(0.20 to 0.58)	<0.001§	N/A
TUG test, *s*‡					<0.001
Month 12	7.54(7.15 to 7.91)	7.89(7.44 to 8.22)	N/A	0.001	N/A
Month 24	7.49(7.19 to 8.03)	7.97(7.53 to 8.34)	N/A	<0.001	N/A
Month 36	7.48(7.07 to 7.95)	8.05(7.64 to 8.45)	N/A	<0.001	N/A
BMD at femoral neck, g/cm^2‡					<0.001
Month 12	0.684(0.657 to 0.705)	0.664(0.648 to 0.674)	N/A	<0.001	N/A
Month 24	0.689(0.661 to 0.702)	0.657(0.639 to 0.665)	N/A	<0.001	N/A
Month 36	0.698(0.673 to 0.710)	0.646(0.629 to 0.657)	N/A	<0.001	N/A
Deoxypyridinoline, *nmol/mmol* ‡					<0.001
Month 12	7.64(6.61 to 8.44)	7.95(6.95 to 8.89)	N/A	0.062	N/A
Month 24	7.47(6.52 to 8.40)	8.20(7.16 to 9.09)	N/A	0.005	N/A
Month 36	7.57(6.61 to 8.37)	8.49(7.38 to 9.31)	N/A	<0.001	N/A
Osteocalcin, *µg/liter* ‡					<0.001
Month 12	12.52(10.50 to 14.81)	11.46(10.21 to 13.58)	N/A	0.091	N/A
Month 24	13.07(10.91 to 15.25)	11.55(10.26 to 13.79)	N/A	0.011	N/A
Month 36	13.59(11.20 to 15.53)	11.71(10.35 to 13.94)	N/A	0.005	N/A

Note: BMD= bone mineral density; TUG=Timed Up and Go, higher scores indicate more severe disease status; BSYJF= Bushen Yijing Fang;

N/A= not appliance.
† Values were presented as mean (95%CI).
‡ Values were presented as median (interquartile range).
* The interaction of treatment group and times were analyzed by Linear generalized estimating equations (GEEs)
**Values were analyzed by Wilcoxon rank-sum test unless otherwise indicated.
§Values were calculated by analysis of covariance model (ANCOVA) at three time points.
The baseline age, time since menopause, body mass index (BMI), and history of falls were used as covariates in Linear GEEs and ANCOVA.

Appendix Table 5 Summary data for changes of 25(OH)D, Endometrial thickness and E2 after 36 months.

Variable	BSYJF(n=70)	Placebo(n=70)	Between-Group Difference		Time-Group Interaction
			BSYJF vs. Placebo	*P* value**	*P* value*
25(OH)D, *ng/mL*†					0.137
Month 12	17.09(16.10 to 18.08)	17.19(16.06 to 18.32)	-0.10(-1.59 to 1.39)	>0.05	N/A
Month 24	16.58(15.57 to 17.59)	16.20(15.06 to 17.34)	0.38(-1.12 to 1.89)	>0.05	N/A
Month 36	15.76(14.70 to 16.83)	15.23(14.06 to 16.39)	0.54(-1.02 to 2.10)	>0.05	N/A
Endometrial thickness, *mm*‡					0.27
Month 12	1.86(1.61 to 2.09)	1.72(1.56 to 1.94)	N/A	>0.05	N/A
Month 24	1.82(1.59 to 2.04)	1.76(1.54 to 1.93)	N/A	>0.05	N/A
Month 36	1.79(1.58 to 2.02)	1.67(1.50 to 1.91)	N/A	>0.05	N/A
E2, *pmol/L*‡					0.53
Month 12	37.38(32.22 to 39.72)	34.15(31.35 to 38.28)	N/A	>0.05	N/A
Month 24	37.23(32.10 to 39.54)	34.79(31.26 to 38.13)	N/A	>0.05	N/A
Month 36	36.35(31.98 to 39.56)	33.73(31.05 to 38.03)	N/A	>0.05	N/A

Note: BSYJF= Bushen Yijing Fang; 25(OH)D= 25-OH-vitamin D; E2= Estradiol; N/A= not appliance.
†Values were presented as mean (95% CI).
‡Values were presented as median (interquartile range).
*The interaction of treatment group and times were analyzed by Linear generalized estimating equations (GEEs)
**Values were analyzed by Wilcoxon rank-sum test unless otherwise indicated.
§Values were calculated by analysis of covariance model (ANCOVA) at three time points.
The baseline age, time since menopause, body mass index (BMI), and history of falls were used as covariates in Linear GEEs and ANCOVA.

Appendix Table 6 Summary data for changes of 25(OH)D after 10-year observational follow-up.

Variable	BSYJF(n=70)	Placebo(n=70)	Between-Group Difference		Time-Group Interaction
			BSYJF vs. Placebo	*P* value**	*P* value*
25(OH)D, *ng/mL*†					0.06
Year 3rd	14.23(13.14 to 15.23)	14.34(13.18 to 15.49)	-0.10(-1.67 to 1.46)	>0.05	N/A
Year 6th	13.47(12.41 to 14.52)	13.59(12.46 to 14.72)	-0.13(-1.65 to 1.40)	>0.05	N/A
Year 10th	12.63(11.67 to 13.59)	12.89(11.78 to 13.99)	-0.26(-1.70 to 1.18)	>0.05	N/A

Note: BSYJF= Bushen Yijing Fang; 25(OH)D= 25-OH-vitamin D; N/A= not appliance.
†Values were presented as mean (95% CI).
*The interaction of treatment group and times were analyzed by Linear generalized estimating equations (GEEs)
**Values were calculated by analysis of covariance model (ANCOVA) at three time points.
The baseline age, time since menopause, body mass index (BMI), and history of falls were used as covariates in Linear GEEs and ANCOVA.

Appendix Table 7 Sensitivity analyses for secondary endpoints.

Variables	BSYJF (70)	Placebo (70)	Between-Group Difference		Time-Group Interaction
			BSYJF vs. Placebo	*P* value**	*P* value*
Lean mass of left thigh, *kg*†					**<0.001**
Month 12	5.53(5.40 to 5.66)	5.37(5.26 to 5.49)	0.17(0.00 to 0.33)	0.040§	N/A
Month 24	5.56(5.43 to 5.69)	5.28(5.18 to 5.39)	0.28(0.11 to 0.44)	0.001§	N/A
Month 36	5.58(5.45 to 5.72)	5.20(5.10 to 5.31)	0.38(0.21 to 0.54)	<0.001§	N/A
TUG test, *s*‡					**<0.001**
Month 12	7.55(7.15 to 7.93)	7.90(7.46 to 8.23)	N/A	0.001	N/A
Month 24	7.49(7.09 to 7.91)	8.00(7.60 to 8.34)	N/A	<0.001	N/A
Month 36	7.44(7.07 to 7.49)	8.07(7.67 to 8.44)	N/A	<0.001	N/A
BMD at femoral neck, *g/cm*^{2}‡					**<0.001**
Month 12	0.664(0.648 to 0.674)	0.684(0.657 to 0.696)	N/A	<0.001	N/A
Month 24	0.686(0.662 to 0.701)	0.657(0.639 to 0.666)	N/A	<0.001	N/A
Month 36	0.694(0.671 to 0.710)	0.646(0.629 to 0.658)	N/A	<0.001	N/A
Deoxypyridinoline, *nmol/mmol*‡					**<0.001**
Month 12	7.59(6.64 to 8.43)	7.98(6.96 to 8.89)	N/A	0.053	N/A
Month 24	7.47(6.59 to 8.39)	8.33(7.22 to 9.12)	N/A	0.001	N/A
Month 36	7.42(6.55 to 8.34)	8.48(7.36 to 9.27)	N/A	<0.001	N/A
Osteocalcin, *μg/liter*‡					**<0.001**
Month 12	12.42(10.40 to 14.81)	11.54(10.22 to 13.67)	N/A	0.135	N/A
Month 24	12.83(10.88 to 15.16)	11.70(10.30 to 13.84)	N/A	0.041	N/A
Month 36	13.05(11.10 to 15.45)	11.80(10.38 to 13.97)	N/A	0.010	N/A
25-OH-vitamin D, *ng/mL*‡					0.125

Month 12	17.13(16.15 to 18.11)	17.26(16.06 to 18.37)	-0.13(-1.57 to 1.31)	>0.05§	N/A
Month 24	16.35(15.39 to 17.31)	16.32(15.25 to 17.38)	0.04(-1.36 to 1.45)	>0.05§	N/A
Month 36	15.53(14.58 to 16.49)	15.49(14.44 to 16.54)	0.06(-1.34 to 1.45)	>0.05§	N/A
Estradiol, *pmol/L*†					0.44
Month 12	37.37(32.29 to 39.69)	34.40(31.36 to 38.19)	N/A	>0.05	N/A
Month 24	37.25(32.17 to 39.55)	34.26(31.26 to 38.09)	N/A	>0.05	N/A
Month 36	37.10(32.05 to 39.46)	34.08(31.13 to 38.01)	N/A	>0.05	N/A
Endometrial thickness, *mm*‡					0.30
Month 12	1.85(1.61 to 2.08)	1.72(1.56 to 1.93)	N/A	>0.05	N/A
Month 24	1.83(1.59 to 2.06)	1.71(1.55 to 1.92)	N/A	>0.05	N/A
Month 36	1.81(1.58 to 2.04)	1.69(1.54 to 1.91)	N/A	>0.05	N/A

Note: BSYJF= Bushen Yijing Fang; BMD= bone mineral density; TUG= Timed Up and Go, higher scores indicate more severe disease status; N/A= not appliance.

†Values were presented as mean (95%CI).

‡Values were presented as median (interquartile range).

*The interaction of treatment group and times were analyzed by Linear generalized estimating equations (GEEs)

**Values were analyzed by Wilcoxon rank-sum test unless otherwise indicated.

§Values were calculated by analysis of covariance model (ANCOVA) at three time points.

Sensitivity analysis using the multiple imputation method estimated the effect of missing values.

The baseline age, time since menopause, body mass index (BMI), and history of falls were used as covariates in Linear GEEs and ANCOVA.

Appendix 2 Methods and Results of Bioinformatics Analysis and *In Vivo* Validation

Methods

The chemical structures, molecular formula, and molecular weight of the compounds in BSYJF were collected and summarized from Traditional Chinese Medicine (TCM) Database (http://tcm.cmu.edu.tw/)[1], Traditional Chinese Medicine systems pharmacology database and analysis platform (TCMSP) Database （http://lsp.nwu.edu.cn/tcmsp.php)[2]. The data of target genes of these postulated compounds were collected from TCMSP database[2]. The functions of target genes were further identified as either bone-dependent or fall-related categories using literature mining by three research fellows with a Ph.D. degree and corresponding research background independently (B.H., B.G. and L.D.). The gene functions that were supported by at least two research fellows were used for further analyses. The interactions between target genes with bone-dependent or fall-related functions (B.H. and B.G.) were identified using literature mining in a similar way as described above. These above postulated chemical compounds in BSYJF were further validated by Ultra Performance Liquid Chromatography and Quadrupole time-of-flight Mass Spectrometry (UPLC-Q-TOF MS) (L.D.). Briefly, all information of compounds in BSYJF was input into the UPLC-Q-TOF MS for finding compounds through their molecular features. The water extraction from BSYJF was run in the UPLC-Q-TOF MS and detected in the full scan mass spectra in both positive ion mode and negative ion mode. The scan results were analyzed by the *Find by Molecular Feature program* to identify the database-matching compounds with targeted molecular formula, molecular weight, as well as the corresponding isotopic pattern and fragmentation profile on the basis of the chemical structures[3].

The herbs in BSYJF were classified into two categories based on the number of target genes in fall-related network, herbs target ≥ 3 genes (HT3G) or herbs target ≤ 2 genes (HT2G). HT3G were proposed as active component. HT3G and HT2G were composed with the original weight ratio in BSYJF. Further animal study was conducted to investigate the fictional roles of these two categories on musculoskeletal system. All protocols and experimental procedures were approved by the Animal Experimentation Ethics Committee of the Chinese University of Hong Kong. All experiments were performed in accordance with the relevant guidelines and regulations outlined in the Animal Experimentation Ethics Committee Guide for the Care and Use of Laboratory Animals, The Chinese University of Hong Kong.

To investigate the role of HT3G within BSYJF in regulating muscle mass and strength, OVX rat model was used. Briefly, thirty-two 6-month old female SD rats were recruited. Ovariectomy was performed to mimic postmenopause. Eight rats were sacrificed as BL group at 6 months post-surgery. The rest rats were assigned into 3 groups as follows, PBS group (n=8): daily oral administration of 2 ml PBS for three months started from 6 months post-surgery; BSYJF group (n=8): daily oral administration of BSYJF powder in PBS (100mg/kg) for three months started from 6 months post-surgery. BSYJF w/o HT3G group (n=8): daily oral administration of HT2G powder in PBS (36mg/kg) for three months started from 6 months post-surgery. All the rats were sacrificed after 3-month intervention. The left EDL was collected for *in vitro* muscle testing and the right EDL was collected for H&E staining to determine the CSA of the muscle fiber.

To investigate the role of HT2G within BSYJF in regulating bone mechanical property, OVX rat model was used. Briefly, thirty-two 6-month old female SD rats were recruited. Ovariectomy was performed to mimic postmenopause. Eight rats were sacrificed as BL at 6 months post-surgery.

The rest rats were assigned into 3 groups as follows, PBS group (n=8): daily oral administration of 2 ml PBS for three months started from 6 months post-surgery; BSYJF group (n=8): daily oral administration of BSYJF powder in PBS (100mg/kg) for three months started from 6 months post-surgery. BSYJF w/o HT2G group (n=8): daily oral administration of HT3G powder in PBS (64mg/kg) for three months started from 6 months post-surgery. All the rats were sacrificed after 3-month intervention. The left femur was collect for three-point bending test. The energy at ultimate force was analyzed.

Results

High consistency in chemical composition from different batches of BSYJF capsules. Total 16 compounds were identified (**Appendix Table 8**) and 20 target genes were found. 5 target genes and 8 interactions fall-related functions, as well as 17 target genes and 51 interactions in bone-dependent functions were found for BSYJF (**Appendix Table 9**). The target genes of BSYJF were significantly enriched in both bone-dependent and fall-related (muscular and neurological functions) signaling pathways (**Appendix Figure 2**).

Based on the number of target genes in fall-related network, Yinyanghuo, Roucongrong, Huangqi, and Gusuibu were classified into HT3G, Heshouwu, Shihu and Juhua were classified into HT2G. In the study of HT3G's role in regulating muscle property, BSYJF treatment maintained mean fiber CSA of EDL in OVX rats, whereas the fiber CSA stayed low after treatment of BSYJF without HT3G. The twitch force of EDL showed similar trend with fiber CSA (**Appendix Figure 3**). In the study of HT2G' role in regulating bone mechanical property, BSYJF treatment maintained the energy at ultimate force of femur in OVX rats, whereas the energy stayed low after treatment of BSYJF without HT2G (**Appendix Figure 4**).

References

1 Chen, C. Y. TCM Database@Taiwan: the world's largest traditional Chinese medicine database for drug screening in silico. *PloS one* **6**, e15939, doi:10.1371/journal.pone.0015939 (2011).

2 Ru, J. *et al.* TCMSP: a database of systems pharmacology for drug discovery from herbal medicines. *Journal of cheminformatics* **6**, 13, doi:10.1186/1758-2946-6-13 (2014).

3 Ferrer, I., Fernandez-Alba, A., Zweigenbaum, J. A. & Thurman, E. M. Exact-mass library for pesticides using a molecular-feature database. *Rapid Commun Mass Spectrom* **20**, 3659-3668, doi:10.1002/rcm.2781 (2006).

Appendix Table 8 Identification of chemical constituents in BSYJF capsules using UPLC-Q-TOF MS in either negative or positive ion modes.

No.	RT (min)	Identification	Ion Mode	MS (*m/z*)	Composition	Herbal Source
1	0.912	Succinic acid	Negative	118.027	$C_4H_6O_4$	RCR
2	4.716	Jaranol	Negative	314.079	$C_{17}H_{14}O_6$	HQ
3	5.309	3,4,3',5'-Tetrahydroxystilbene-3-glucoside	Negative	406.126	$C_{20}H_{22}O_9$	HSW
4	5.486	Astragalin	Positive/Negative	488.1	$C_{21}H_{20}O_{11}$	GSB
5	7.753	Nodakenetin	Positive	246.089	$C_{14}H_{14}O_4$	SH
6	7.8	Icaride A2	Positive	436.172	$C_{22}H_{28}O_9$	YYH
7	7.849	Daidzein	Negative	254.058	$C_{15}H_{10}O_4$	HQ
8	7.892	Icariside A7	Negative	462.152	$C_{23}H_{26}O_{10}$	YYH
9	8.136	Eriodictyol	Negative	288.063	$C_{15}H_{12}O_6$	GSB
10	8.729	Borneol	Negative	154.136	$C_{10}H_{18}O$	JH
11	11.541	Formononetin	Negative	268.073	$C_{16}H_{12}O_4$	HQ
12	12.954	Soyasaponin I	Negative	942.517	$C_{48}H_{78}O_{18}$	HQ
13	14.906	Genistein	Negative	270.053	$C_{15}H_{10}O_5$	RCR
14	16.351	Bilobanol	Negative	234.162	$C_{15}H_{22}O_2$	YYH
15	16.415	Alpha-Curcumene	Negative	202.171	$C_{15}H_{22}$	JH
16	16.768	Suchilactone	Negative	368.123	$C_{21}H_{20}O_6$	RCR

Note: BSYJF= Bushen Yijing Fang; ESI= Electrospray ionization; RT= retention time. RCR= Roucongrong; HQ= Huangqi; HSW= Heshouwu; GSB= Gusuibu; SH= Shihu; YYH= Yinyanghuo; JH= Juhua.

Appendix Table 9 Bone-dependent and fall-related functions of target genes of chemical constituents in BSYJF.

Function	Target Gene	Compound	Herb
BD	CCL2	Genistein	RCR
BD	FN1	Genistein	RCR
BD	FOS	Daidzein	HQ
BD	FOS	Genistein	RCR
BD	ICAM1	Daidzein	HQ
BD	ICAM1	Genistein	RCR
BD	IL1B	Genistein	RCR
BD	IL6	Daidzein	HQ
BD	JUN	Daidzein	HQ
BD	JUN	Formononetin	HQ
BD	JUN	Genistein	RCR
BD	LDLR	Daidzein	HQ
BD	LDLR	Genistein	RCR
BD	MAPK1	Genistein	RCR
BD	MAPK14	Astragalin	GSB
BD	MAPK14	Daidzein	HQ
BD	MAPK14	Eriodictyol	GSB
BD	MAPK14	Formononetin	HQ
BD	MAPK14	Genistein	RCR
BD	MAPK14	Icaride A2	YYH
BD	MAPK14	Jaranol	HQ
BD	MAPK14	Suchilactone	RCR
BD	NOS2	Borneol	JH
BD	NOS2	Astragalin	GSB
BD	NOS2	Bilobanol	YYH
BD	NOS2	Daidzein	HQ
BD	NOS2	Eriodictyol	GSB
BD	NOS2	Formononetin	HQ
BD	NOS2	Genistein	RCR
BD	NOS2	Icaride A2	YYH
BD	NOS2	Jaranol	HQ
BD	NOS2	Nodakenetin	SH
BD	NOS2	Succinic acid	RCR
BD	NOS2	Suchilactone	RCR
BD	PPARG	Daidzein	HQ
BD	PPARG	Formononetin	HQ
BD	PPARG	Genistein	RCR
BD	RHOA	Daidzein	HQ
BD	TGFB1	Genistein	RCR
BD	VEGFA	Daidzein	HQ
BD	VEGFA	Genistein	RCR

FR	ADRA2B	Nodakenetin	SH
FR	GSK3B	Astragalin	GSB
FR	GSK3B	Eriodictyol	GSB
FR	GSK3B	Formononetin	HQ
FR	GSK3B	Icaride A2	YYH
FR	GSK3B	Jaranol	HQ
FR	GSK3B	Suchilactone	RCR
FR	NR3C1	Soyasaponin I	HQ
FR and BD	PTGS2	Borneol	JH
FR and BD	PTGS2	3,4,3',5'-Tetrahydroxystilbene-3-glucoside	HSW
FR and BD	PTGS2	Alpha-Curcumene	JH
FR and BD	PTGS2	Astragalin	GSB
FR and BD	PTGS2	Daidzein	HQ
FR and BD	PTGS2	Eriodictyol	GSB
FR and BD	PTGS2	Genistein	RCR
FR and BD	PTGS2	Icaride A2	YYH
FR and BD	PTGS2	Icariside A7	YYH
FR and BD	PTGS2	Jaranol	HQ
FR and BD	PTGS2	Nodakenetin	SH
FR and BD	PTGS2	Suchilactone	RCR
FR and BD	TNF	Daidzein	HQ
FR and BD	TNF	Genistein	RCR

Note: BSYJF= Bushen Yijing Fang; FR= Fall-related; BD= Bone-dependent; RCR= Roucongrong; HQ= Huangqi; GSB= Gusuibu; YYH= Yinyanghuo; SH=Shihu; HSW= Heshouwu; JH=Juhua.

A.

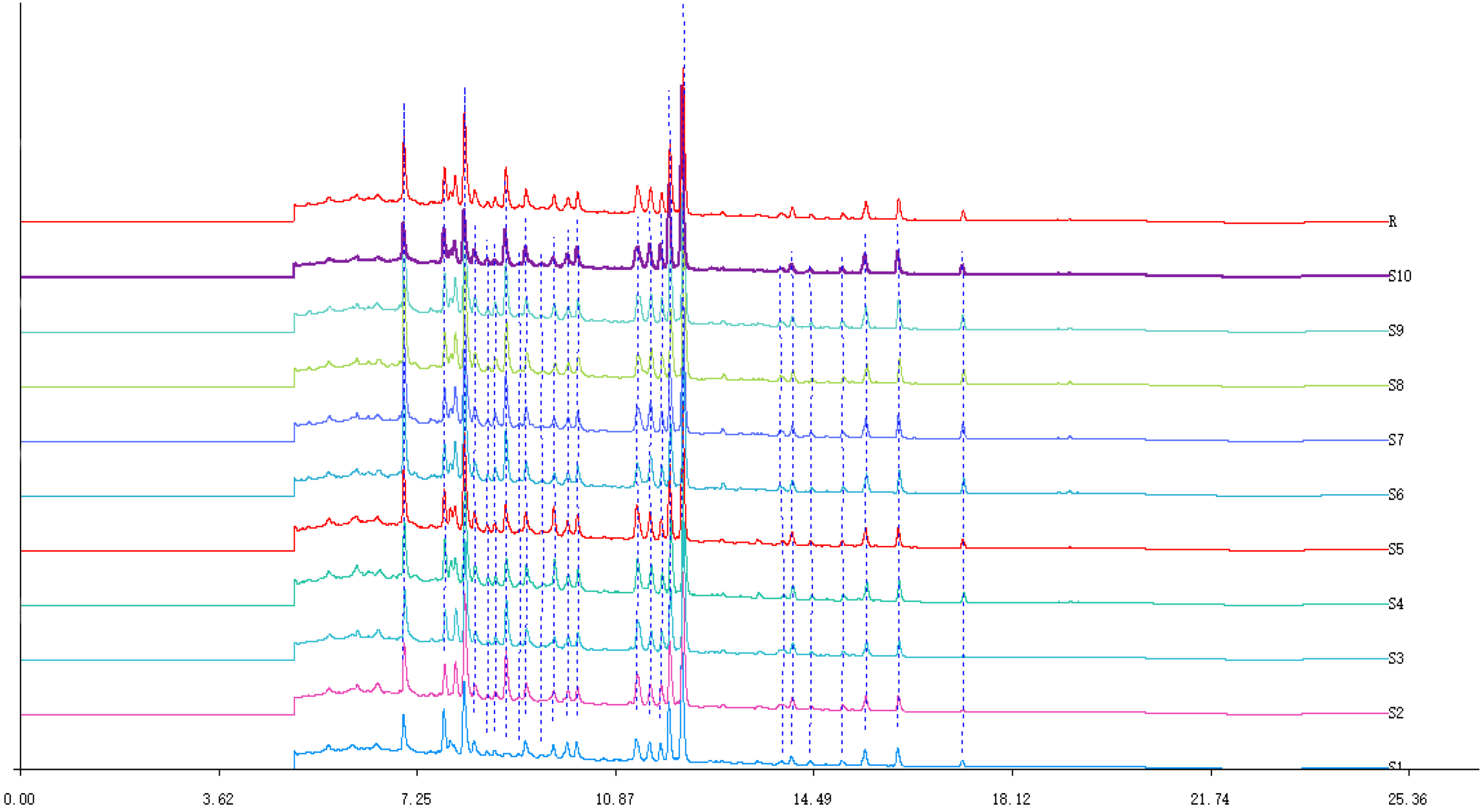
R
S10
S9
S8
S7
S6
S5
S4
S3
S2
S1
0.00
3.62
7.25
10.87
14.49
18.12
21.74
25.36

B.

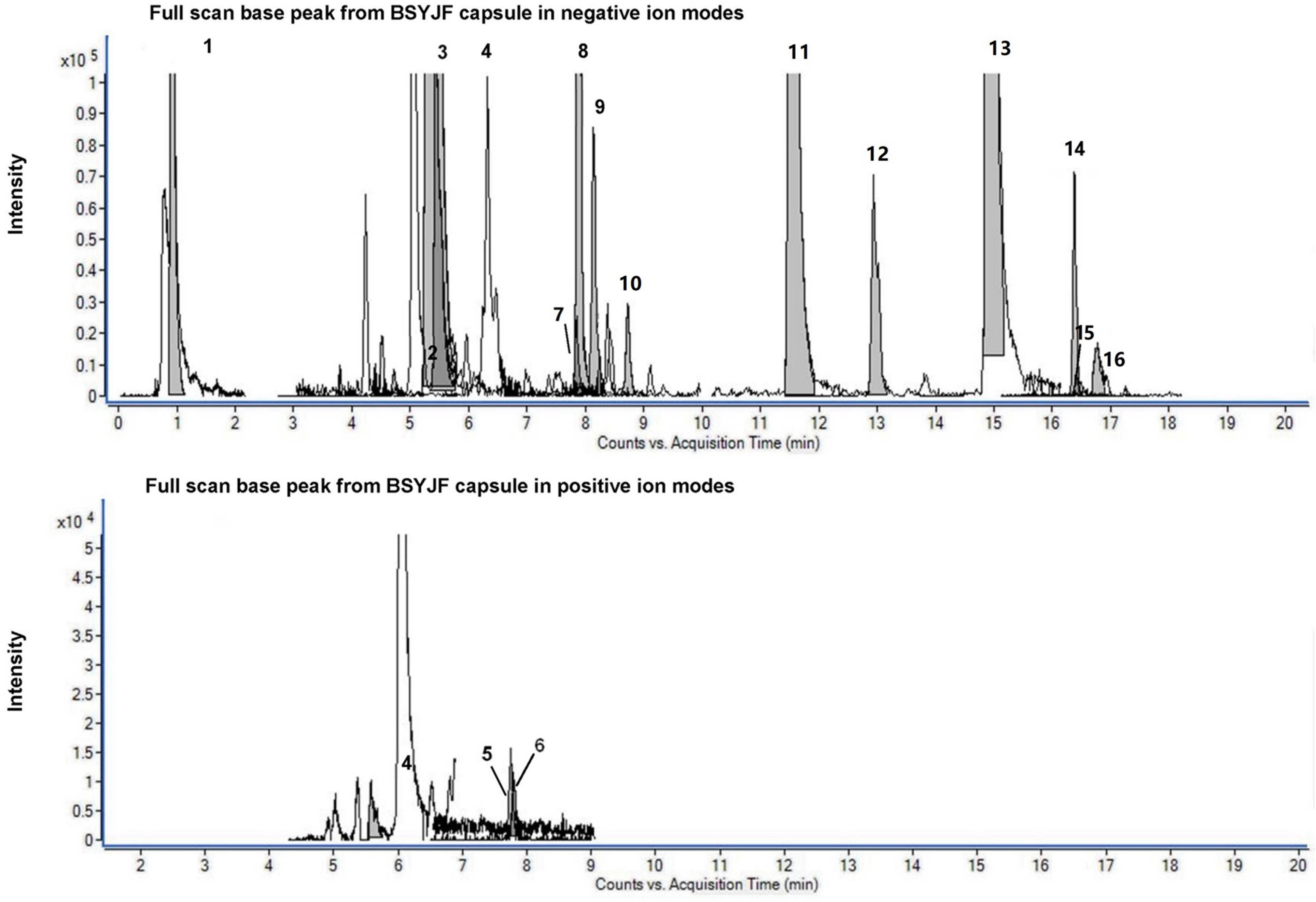
Full scan base peak from BSYJF capsule in negative ion modes
x10 5
Intensity
Counts vs. Acquisition Time (min)
1
2
3
4
7
8
9
10
11
12
13
14
15
16
Full scan base peak from BSYJF capsule in positive ion modes
x10 4
Intensity
Counts vs. Acquisition Time (min)
4
5
6

C.

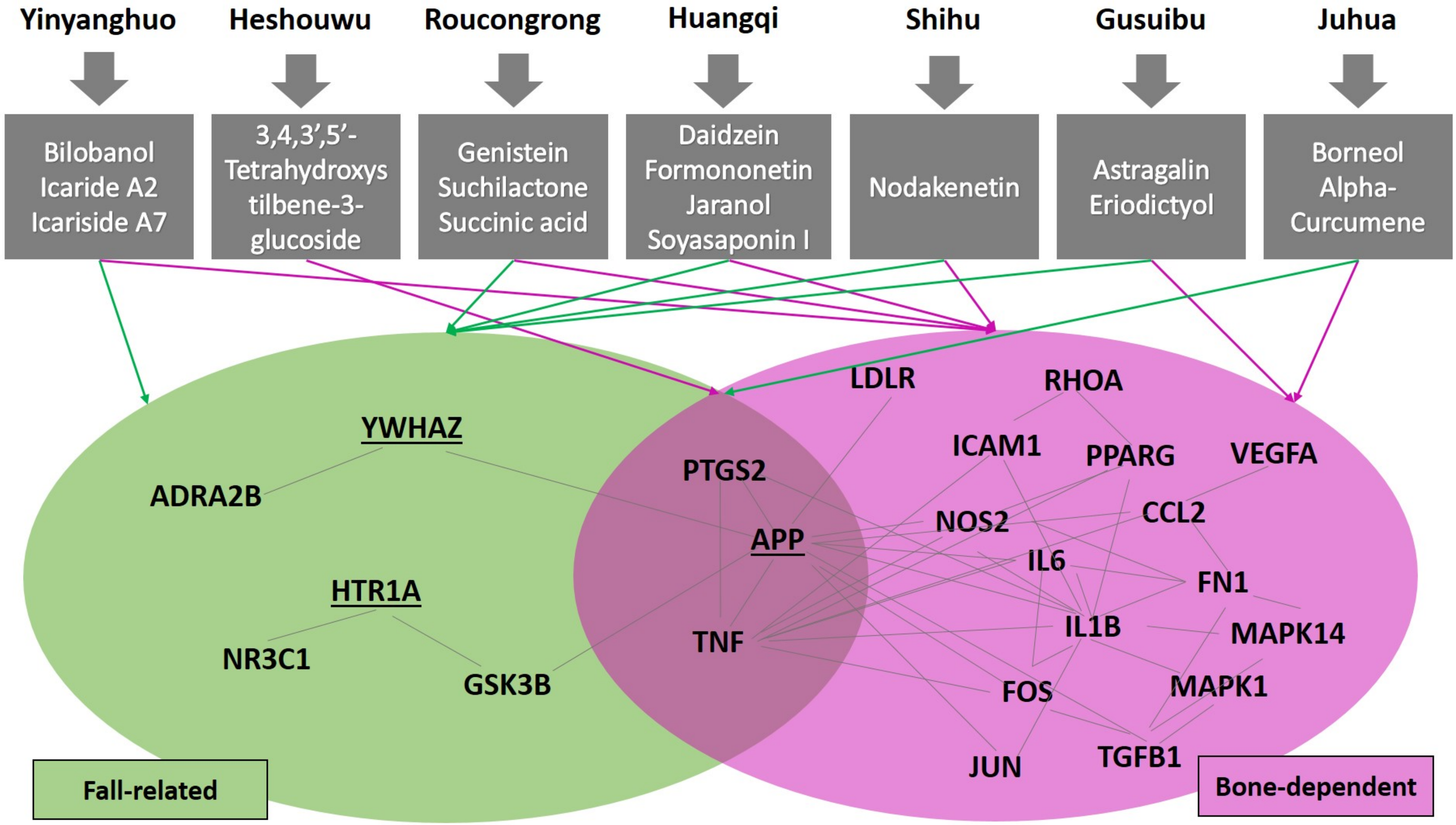
Yinyanghuo
Heshouwu
Roucongrong
Huangqi
Shihu
Gusuibu
Juhua
Bilobanol
Icaride A2
Icariside A7
3,4,3',5'-Tetrahydroxystilbene-3-glucoside
Genistein
Suchilactone
Succinic acid
Daidzein
Formononetin
Jaranol
Soyasaponin I
Nodakenetin
Astragalin
Eriodictyol
Borneol
Alpha-Curcumene
YWHAZ
ADRA2B
HTR1A
NR3C1
GSK3B
PTGS2
APP
TNF
LDLR
RHOA
ICAM1
PPARG
VEGFA
NOS2
CCL2
IL6
FN1
IL1B
MAPK14
FOS
MAPK1
JUN
TGFB1
Fall-related
Bone-dependent

Appendix Figure 2 Bioinformatic analysis results. (A) Chromatographic profiles of BSYJF from different batches of capsules by HPLC-UV. **(B)** Full scan base peak chromatogram of methanol extract from BSYJF capsule in either negative or positive ion modes by UPLC-Q-TOF MS. The labels of the peak represented BSYJF compound in **Appendix Table 8**. **(C)** Bone-dependent and fall-related target genes of the compounds detected from BSYJF capsule. The underlined genes were connecting genes in interactions instead of target genes. **Note:** BSYJF= Bushen Yijing Fang; HPLC-UV= High Performance Liquid Chromatography-ultraviolet; UPLC-Q-TOF MS= Ultra Performance Liquid Chromatography and Quadrupole time-of-flight Mass Spectrometry. R: Reference chromatogram obtained by average chromatography profiles of tested batches.

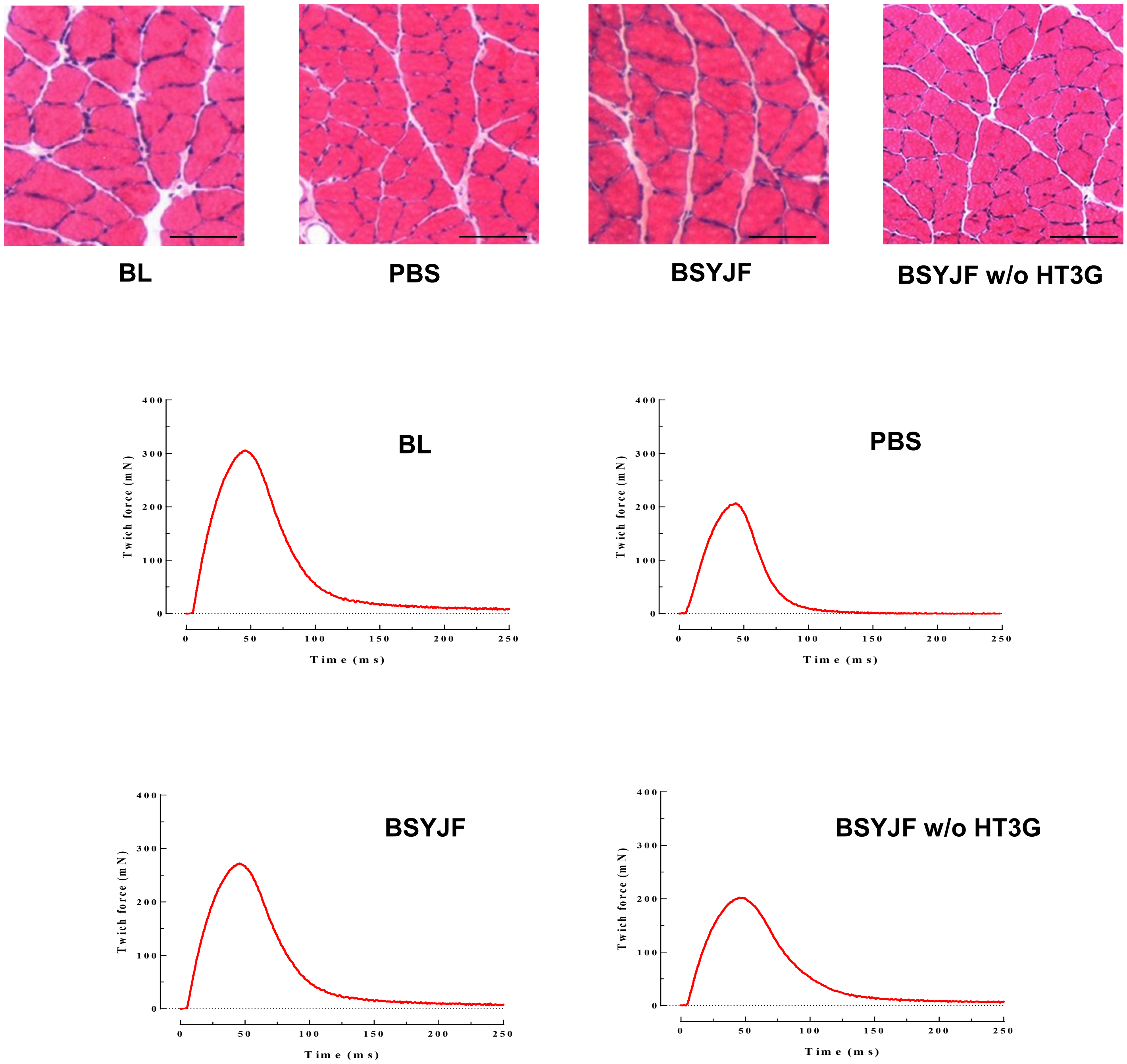

Appendix Figure 3 Role of HT3G within BSYJF in regulating muscle mass and strength in OVX rat. Representative H&E staining of muscle fiber in extensor digitorum longus (EDL) from indicated groups (bar=50μm) (top). Muscle twitch force of EDL from indicated groups (bottom). **Note:** BL= Baseline; BSYJF= Bushen Yijing Fang. HT3G= Herbs target ≥ 3 genes.

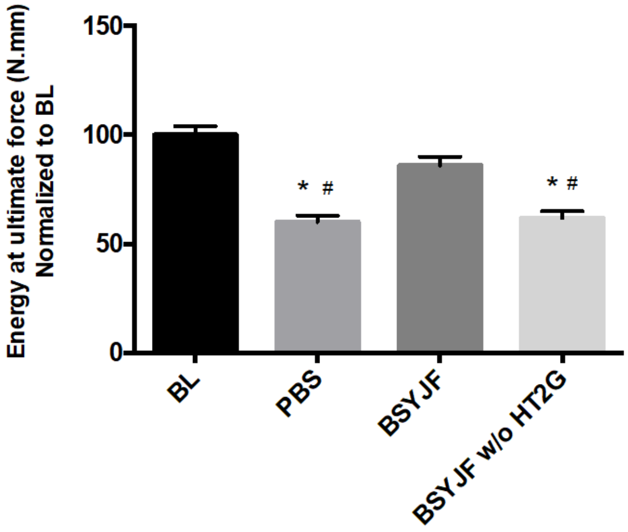

Appendix Figure 4 Role of HT2G within BSYJF in regulating bone mechanical property in OVX rat. Energy at ultimate force of femur from OVX rats determined by three-point bending. **Note:** BL= Baseline; BSYJF= Bushen Yijing Fang. HT2G= Herbs target ≤ 2 genes. *P<0.05 vs. BL; #P<0.05 vs. BSYJF.
