# Supplementary material for: Bushen Yijing Fang Reduces Fall Risk in Late Postmenopausal Women with Osteopenia: A Randomized Double-blind and Placebo-controlled Trial
Source: Sci Rep. 2019 Feb 14;9:2089. doi: 10.1038/s41598-018-38335-3 (PMC6375933; doi:10.1038/s41598-018-38335-3)
Supplement: Supplementary file 2 — Protocol [file 41598_2018_38335_MOESM2_ESM.pdf]

Protocol

Protocol for: Bushen Yijing Fang Reduces Fall Risk in Late Postmenopausal Women with Osteopenia: A Randomized Double-blind and Placebo-controlled Trial

This supplement contains: Original protocol, amendments, and summary of amendments

The effects and safety of Bushen Yijing Fang reduces hip fractures risk factors in late postmenopausal women with osteopenia: A long-term follow-up, randomized, double-blind and placebo-controlled trial

Study leader: Shi Yinyu
Zhang Ge
Zheng Yuxin
Study leader's address： 185 Pu'an Road, Shanghai, China

Study leader's telephone: 53821650

Institutions: 1. Shanghai Shuguang Hospital
2. Orthopaedics and Traumatology Institute with Shanghai Academy of Traditional Chinese Medicine

Version number: Ver1.0

Version Date: August 1999

Funding by the Ministry of Science & Technology of China (Project No. 96-906-09-05), and Shanghai Science & Technology Commission (Project No. 004319226).

Investigator's Agreement

1. Sponsor

I will be in accordance with the requirement of "Good Clinical Practice (GCP) for Quality Control of Drug Clinical Trials", to fulfill the duties of the sponsor conscientiously, including sponsoring, organizing, coordinating and monitoring this clinical study, in particular insurance coverage for all the research-related injuries or deaths, all the costs of treatment and financial compensation, and the provision of law to the investigators and subjects.

Sponsor: Shanghai Shuguang Hospital

Person-in-charge: Shi Yinyu Date: 10 September 1999

2. Investigator

I will carry out the clinical trial in accordance with requirements of this Standard Operating Procedure (SOP) program, and "Good Clinical Practice for Quality Control of Drug Clinical Trials".

I will record the trial data according to the procedures in the Chinese GCP regulations, and ensure that the data in the Case Report Form (CRF) in a true, accurate and timely manner. I agree to accept the sponsor to manage and inspect to ensure the quality of clinical trial.

I will be responsible for making trial-related medical decisions to ensure that subjects will receive the treatment as soon as possible if adverse events occur during the trial. I understand the procedures and requirements for reporting of adverse events properly. If adverse events or serious adverse events were found in the study, I would report according to the protocol as requested.

Institute: Department of Orthopaedics and Traumatology, Shanghai Shuguang Hospital

Person-in-charge: Zheng Yuxin Date: 10 September 1999

Institute: Orthopaedics and Traumatology Institute with Shanghai Academy of Traditional Chinese Medicine

Person-in-charge: Zhang Ge Date: 10 September 1999

3．Principal Inspector

I will fulfill the responsibilities of the auditors in accordance with “Good Clinical Practice for Quality Control of Drug Clinical Trials”.

Institute：Office of Scientific Research and Teaching, Shanghai Shuguang Hospital

Person-in-charge: Zhang Huaiqiong Date: 10 September 1999

4．Statistician

I will fulfill the responsibilities of the statistical qualifications in accordance with the “Good Clinical Practice for Quality Control of Drug Clinical Trials ".

Statistical unit: Department of Statistics, Shanghai University of Traditional Chinese Medicine

Person-in-charge: Shi Meiyu Date: 10 September 1999

Table of Contents

Page

1. STUDY GLOSSARY 8

2. PROTOCOL SYNOPSIS 9

3. **BACKGROUND AND RATIONALE** 11

3.1 Hip fractures 11

3.2 BSYJF Background 11

3.3 Rationale 12

4. STUDY PURPOSE 12

5. TRIAL PLAN 12

5.1 Study Design 12

5.2 Study Ethics 13

6. SUBJECT ELIGIBILITY 13

6.1 Inclusion Criteria 13

6.2 Exclusion Criteria 13

7. SUBJECT ENROLLMENT PROCEDURES 133

7.1 Study Visit Definitions 13

7.2 Methods of Enrollment 14

7.3 Informed Consent 14

7.4 Screening and Baseline Assessment 14

8. RANDOMIZATION AND BLINDING 15

8.1 Randomization 15

8.2 Blinding 16

9. DRUG ADMINISTRATION 16

9.1 Packaging and Formulation 16

9.2 Labeling and Storage 16

9.3 Administration 16

10. QUALITY CONTROL OF TEST CAPSULES 17

10.1 BSYJF Capsules 17

10.2 Placebo Capsules 17
11. CALCULATION OF SAMPLE SIZE 17
12. MEASUREMENTS 18
12.1 BMD and Muscle Measurement 18
12.2 TUG Test 18
12.3 Falls Recording 19
12.4 Laboratory Assessments 19
12.5 Uterus Endometrial Thickness Monitoring 20
13. SAFETY DATA REPORTING 20
13.1 Adverse Events Definitions 20
13.2 Serious Adverse Events 21
13.3 Adverse Event Collection and Reporting 21
13.4 Safety Monitoring 22
14. DROPOUT 22
15. ENDPOINTS 22
15.1 Primary Endpoints 22
15.2 Secondary Endpoints 23
16. STATISTICAL CONSIDERATIONS 23
16.1 Data Collection Forms 23
16.2 Data Management 23
16.3 Statistical Analysis 23
17. QUALITY ASSURANCE 24
17.1 Training 24
17.2 Diet and Exercise Control 24
17.3 Data Quality 24
17.4 Monitoring 25
17.5 Interim Analysis and Stopping Guidelines 25
18. PARTICIPANTS' RIGHTS AND CONFIDENTIALITY 25
18.1 Institutional Review Board 25
18.2 Subject Confidentiality 26

18.3 Conflict of Interests 26

19. PUBLICATION POLICY 26

20. FUNDING 26

21. REFERENCES 27

22. APPENDICES 29

Appendix A. Human Ethics Approval 29

Appendix B: Patient Information Sheet and Informed Consent Form 30

Appendix C. Falls Recording Form 39

Appendix D. Timed Up and Go (TUG) test 40

Appendix E. Adverse Event Severity Scoring System 41

Appendix F. Questionnaire for Daily Dietary Intake and Physical Activities 42

23. AMENDMENTS 1: 43

24. AMENDMENTS 2: 50

25. SUMMARY OF AMENDMENTS 57

1. STUDY GLOSSARY

Abbreviation or Term	Definition/Explanation
GCP	Good Clinical Practice
GMP	Good Manufacturing Practice
CRF	Case Report Form
SAE	Serious Adverse Event
SOP	Standard Operating Procedure
BMD	Bone Mineral Density
ECG	Electrocardiogram
SD	Standard Deviation
ALT	Alanine Aminotransferase
AST	Aspartate Aminotransferase
TCM	Traditional Chinese Medicine
Cr	Creatinine
ICF	Informed Consent Form
IRB	Institutional Review Board
PI	Principal Inspector
CFDA	Chinese State Food and Drug Administration
DXA	Dual X-ray Absorptiometry
BSYJF	Bushen Yijing Fang
DDIPA	Questionnaire for daily dietary intake and physical activities
TUG	Timed Up and Go Test
ITT	Intent to Treat
HPLC	High-performance Liquid Chromatography
ANOVA	Analysis of Variance
LOCF	Last Observation Carry Forward

2. PROTOCOL SYNOPSIS

Protocol name	The effects and safety of Bushen Yijing Fang (BSYJF) reduces hip fractures risk factors in late postmenopausal women with osteopenia: A long-term follow-up, randomized, double-blind and placebo-controlled trial
Sponsor	Shanghai Shuguang Hospital
Indication	Late postmenopausal women with osteopenia
Purpose	To evaluate long-term efficacy of BSYJF on hip fracture risk factors in late postmenopausal women with osteopenia.
Design	Randomized, 36-month, double-blind and placebo-controlled trial
Simple size	140 subjects (70 per group)
Setting	Two sites
Intervention time	From 2000-01-01 To 2004-12-31
Eligibility criteria	<u>Inclusion:</u><br>1. Women with at least 10 years natural menopause, and aged from 55 to 69 years;<br>2. Osteopenia at least 12 months (T-score between −2.5 and −2 SD at femoral neck).<br><br><u>Exclusion:</u><br>1. Who had been diagnosed as having a neurological or musculoskeletal disorder, or coexisting chronic diseases;<br>2. Who have taken estrogen and calcitonin, fluoride, bisphosphonates, or adrenocortical hormone within one year;<br>3. Who had been took ≥ 4 prescription medications;<br>4. has environmental hazards for falls or tripping;<br>5. has impairment in gait;<br>6. has postural hypotension: drop in systolic blood pressure ≥20mmHg or to <90mmHg on standing;<br>7. has impairment in transfer skills or balance;<br>8. has impairment in leg or arm muscle strength or range of motion (hip, ankle, knee, shoulder, hand, elbow) ;

	9. has ALT or AST levels greater than 50% of upper normal limit; serum creatinine levels greater than 133μmmol/l or 1.5 mg/dl.
Drug administration	300mg element calcium daily with either BSYJF capsules (3 capsules per time, 3 times per day) or placebo.
Study period	36-month
Endpoints	Endpoints are to be evaluated for BSYJF versus placebo.<br>Primary endpoints:<br>1. Change from month baseline at month 36 in femoral neck BMD;<br>2. Change from month baseline at month 36 in lean mass of left thigh;<br>3. Change from month baseline at month 36 in TUG test;<br>4. The number of falls at month 36.<br><br>Secondary endpoints:<br>1. Change from month baseline at month 36 in biomarkers of bone turnover (osteocalcin and deoxypyridinoline).<br>2. Change from month baseline at month 36 in endometrial thickness;<br>3. Change from month baseline at month 36 in estradiol;<br>4. Nature, frequency and severity of adverse events and their relationship to treatment.
Statistical considerations	All the analyses will use randomly assigned patients under ITT principle. For patients missing data, the LOCF approach use to analyze the longitudinal data. Repeated-measures ANOVA analysis should be carried out between two groups and four times (baseline, 12, 24 and 36 months). Within-subjects results will be used *t*-test on the percentage change from the baseline value. The number of falls and adverse events will perform by chi-square test. All tests will be used two-sided and set at the 5% level.

3. BACKGROUND AND RATIONALE

3.1 Hip fractures

Hip fractures cause disability and even mortality in community-dwelling elderly women, which is a devastating consequence of osteoporosis (Cooper and Barker, 1995; Zuckerman, 1996). Although many factors contribute to such fractures, the most important causes are a reduction in bone mass and an increased frequency of falls (Chapuy and Arlot et al., 1992). It is estimated that approximately 90% hip fractures in late postmenopausal women resulted from a fall (Zuckerman, 1996). It has been known that the deteriorated muscle and impaired functional mobility increases the falling risk factors obviously (Greenspan and Myers et al., 1994). Thus, it indicates that the bone-dependent risk factors including decreased bone mass and deteriorated bone micro-architecture are not the only determinant of hip fracture. In other words, the bone-independent risk factors including decreased muscle and functional mobility also play an important role in hip fracture risk factors in late postmenopausal women.

The conventional medications for preventing hip fractures only focus on bone-dependent risk factors by either inhibiting bone resorption (bisphosphonates) or promoting bone formation (rhPTH). Despite that supplement of vitamin D has demonstrated beneficial effects on muscle, there is still lack of high quality evidence to support that vitamin D alone could prevent hip fractures effectively, especially for the late postmenopausal women with osteopenia (Chapuy and Arlot et al., 1992). Therefore, it is desirable to develop alternative medications that can exert beneficial effects on reducing both bone-dependent and bone-independent risk factors.

3.2 BSYJF Background

The Chinese herbal formula, such as BSYJF, has a long history of application to disease treatment for musculoskeletal disorders with “bone-invigorating” and “muscle-strengthening” functions in China. BSYJF is composed of 7 herbs, *Herba Epimedii* (Yinyanghuo in Chinese), *Radix Polygoni Multiflori* (Heshouwu in Chinese), *Herba Cistanches* (Roucongrong in Chinese), *Radix Astragali* (Huangqi in Chinese), *Rhizoma Drynariae* (Gusuibu in Chinese), *Herba Dendrobii* (Shihu in Chinese) and *Flos Chrysanthemi* (Juhua in Chinese) with a crude weight ratio in 10:6:6:10:6:6:6.

Animal studies support the notion that BSYJF could delay bone loss of femoral

cortical and cancellous bone with aging, improve the structure of the bone matrix, the area and depth of resorption lacunae and bone strength, inhibit osteoclasts desorption, increase trabecular thickness as well as osteoblast number in ovariectomy (OVX) or glucocorticoid -induced osteoporosis model. BSYJF also exerts its beneficial effect on muscles of low extremity (Song and Shi et al., 1995; Song and Shi et al., 1996; Shen and Xu et al., 1997; Song and Shi, 1997; Song and Song et al., 1997; Song and Shen et al., 1998; Zhang and Shi et al., 1999; Zhang and Shi et al., 1999).

Among BSYJF in the treatment of osteoporosis, total efficacy rate ((Total symptom score at baseline - Total symptom score post treatment) / Total symptom score at baseline ×100%) is 67.31%, and BMD is maintained in our precious observation (Song and Song et al., 1997).

3.3 Rationale

Recently, the BSYJF has been prescribed in the treatment of osteoporosis in postmenopausal women. Moreover, those component herbs in BSYJF have been also prescribed by Chinese Medicine Practitioners in the treatment of musculoskeletal disorders through the centuries. However, the long-term effects of BSYJF on preventing falls and hip fractures associated risk factors have not been established. In this study, we will perform a double-blind and placebo-controlled trial to evaluate the long-term effect of BSYJF on reducing bone-dependent risk factor (bone mass) and bone-independent risk factors (muscle mass and functional mobility) to prevent falls and hip fractures risk factors in late postmenopausal women with osteopenia.

4. STUDY PURPOSE

To assess long-term effects of BSYJF on hip fractures risk factors in treating late postmenopausal women with osteopenia.

5. TRIAL PLAN

5.1 Study Design

The study duration is from January 2000 to December 2004. Subjects will be randomly assigned to receive BSYJF (n=70) or placebo (n=70) with a 1:1 allocation ratio. The study will be carried out at the two sites (Orthopaedics and Traumatology Institute with Shanghai Academy of Traditional Chinese Medicine, and Shuguang Hospital). A total of 140 participants will be selected from two sites to participate in the study.

5.2 Study Ethics

This study complies with the principles of the Declaration of Helsinki. This trial is approved by IRB of Orthopaedics and Traumatology Institute with Shanghai Academy of Traditional Chinese Medicine (Appendix A).

6. SUBJECT ELIGIBILITY

6.1 Inclusion Criteria

(1) Women with at least 10 years after natural menopause, and aged from 55 to 69 years;

(2) Osteopenia at least 12 months (T-score between −2.5 and −2 SD at femoral neck).

6.2 Exclusion Criteria

The postmenopausal women will be excluded from this study if:

(1) Who had been diagnosed as having a neurological or musculoskeletal disorder, or coexisting chronic diseases;

(2) Who have taken estrogen and calcitonin, fluoride, bisphosphonates, or adrenocortical hormone within one year;

(3) Who had been took ≥ 4 prescription medications;

(4) has environmental hazards for falls or tripping;

(5) has impairment in gait;

(6) has postural hypotension: drop in systolic blood pressure ≥20mmHg or to <90mmHg on standing;

(7) has impairment in transfer skills or balance;

(8) has impairment in leg or arm muscle strength or range of motion (hip, ankle, knee, shoulder, hand, or elbow) ;

(9) has ALT or AST levels greater than 50% of upper normal limit; serum creatinine levels greater than 133μmmol/l or 1.5 mg/dl.

7. SUBJECT ENROLLMENT PROCEDURES

7.1 Study Visit Definitions

Screening date is defined as the date that the ICF is signed. Enrollment date is defined as the date of randomization. The randomization must be completed within 2 weeks of the screening date (or re-screened within 2 weeks). If a subject’s visit is delayed, her subsequent visit should not be shifted.

7.2 Methods of Enrollment

The recruitment goal is a total sample size of 140 participants. Researchers

at each site will identify qualified participants for the trial from clinical records based on the specified inclusion and exclusion criteria. Medical record reviews of existing databases can be done initially by setting up the searches using the matched eligible criteria. Potential participants will be also contacted by outreach materials or approached person at the clinic to determine their interest in the study. They will be briefly told about the study, and they will be invited for screening and informed consent if interested.

7.3 Informed Consent

Before subjects may be entered into the trial, the investigator requires a copy of the site's written IRB approval of the protocol. A single participant information sheet and consent form will be used for all the procedures done as part of the screening, baseline and follow-up (Appendix B).

The information sheet and consent form will be given to the potential participant and consent details will be explained, including trial's purpose, screening and study procedures, benefits and risks, confidentiality, rights, and trial contact information. The investigator obtaining consent will ask questions to the participant about her correct understanding the consent. The participant will be given the opportunity to review the document and any questions or concerns will be addressed. The participation is free of coercion or intimidation and no material compensation. All participants will be ascertained that they have the right to discontinue voluntarily from the study at any time, without penalty or loss of entitled benefits. If the participant agrees to take part in the trial, two copies of a signed consent form will be made, with the signatures of the participant and the site investigator. One copy of the consent form will be given to the individual to keep and another copy will be kept in a locked storage. For participants who are illiterate, the third party witness must sign ICF.

These consent procedures has been reviewed and approved by IRB. The Office of Scientific Research and Teaching will ensure proper collection and the ICF will be stored in a secure location.

7.4 Screening and Baseline Assessment

After ICF has been obtained at the first pre-randomization visit, the consented participant will undergo initial screening which will include basic medical and

medication history, and hip BMD measurement.

At the end of the first pre-randomization visit, the consented participant will be scheduled for a second visit within about 1 week for screening. The participant will be asked to do transvaginal ultrasound and blood test to chec endometrial thickness, and kidney and liver function, etc. A venous blood sample (approx. 10 ml) will be taken for blood biochemistries and a urine sample to determine if the individual meets eligibility criteria. A screening log will be recorded by each site to each candidate.

The assessment will be included:

- Medical and medication history(including history of falls);
- Physical examination (including height and weight);
- 12-lead ECG;
- Vital signs: blood pressure, temperature, pulse and heart rate;
- DXA scan of proximal femur;
- Samples: blood count, liver and kidney function, 25 (OH) vitamin D, osteocalcin, 17β-estradiol and urine deoxypyridinoline;
- Safety.

At the third visit, those participants who meet the eligibility criteria will be enrolled in the trial and continue with a further baseline assessment and randomization.

All participates who enter into the screening period will receive a unique subject identification number. Participates numbers will be assigned in sequential order. The subject identification number must remain constant throughout the clinical trial, that's to say, it must not be changed at the time of re-screening, enrollment, or randomization. This number will not be the same as the randomization number assigned for the study. All the participants must be subjected to physical examination, efficacy and safety assessment during the recruitment.

8. RANDOMIZATION AND BLINDING

8.1 Randomization

All the participants will be randomly assigned to either BSYJF or placebo. Randomization numbers will be generated with random number tables by an independent statistician from the department of statistics, Shanghai University

of Traditional Chinese Medicine. This statistician is not involved in following data collection or analysis. The codes will be concealed in sealed, opaque envelopes with date and signature labels.

8.2 Blinding

The blinding codes will be given to the pharmacist to keep and cannot be broken unless in emergency situations during the study. The treatment arrangements will be made by the pharmacist in each site, be blinded to the participants' characteristics and be not involved in the number generation and recruitment process.

9. DRUG ADMINISTRATION

9.1 Packaging and Formulation

All the participants will be received daily calcium carbonate 300 mg as the basic treatment. The participants will be orally given BSYJF capsules or placebo capsules (3 capsules per time, 3 times per day) for 36 months. All the capsules should package in identical plastic bottles (100 capsules per bottle) and subsequently delivered to the participants every 3 month at each clinical visit. All the participants will be told to take the capsules with element calcium before meal. The quality control for screening and verification of adherence to the protocol will perform by the supervisor. BSYJF/placebo will be manufactured, packaged, and distributed by Shuguang Hospital.

9.2 Labeling and Storage

BSYJF/placebo will be properly labeled with the product name, investigational product herbs, appropriate cautionary statement, and appropriate storage conditions. Label information for study medication boxes will comply with GCP requirements and a tag indicating 'for trial use only' will be marked.

BSYJF/placebo should be stored in the normal temperature and protected from light. Exposure to higher temperatures and vigorous shaking can lead to a loss of activity and should be avoided. The storage conditions for BSYJF/placebo must be maintained during the period of the study.

9.3 Administration

Detailed records of the case must be administered (date, time, and dose dispensing signature) and unused study medication to inventory count. If a subject misses a scheduled dose of capsules, the dose should be taken as

soon as possible as long as it is before the next scheduled dose, and should be resumed at the same dose. Double or extra doses should not be taken.

10. QUALITY CONTROL OF TEST CAPSULES

10.1 BSYJF Capsules

The whole BSYJF capsules processes should be produced in compliance with standards of GMP and Chinese Pharmacopoeia. Firstly, all herbal medicines will be tested in accordance with the Chinese pharmacopoeia before the production. Then, these 7 herbs will be mixed in above proportion, extracted in water, crushed into powder, and then packed into capsules (0.55g/capsule). Finally, the BSYJF capsules will be examined for the contamination with heavy metals, toxic elements, microbes and pesticide residues. The main active component in BSYJF, icariin ($C_{33}H_{40}O_{15}$), should be validated and quantified by HPLC after production to confirm its pharmaceutical stability in different batches.

10.2 Placebo Capsules

Placebo requires no active components, which will not be used for reproducing the real efficacy of BSYJF. Therefore, mimicking the original color and taste of the BSYJF without adding any active components is a difficult procedure. The main ingredients in placebo capsules are starch supplemented with food colorants and flavoring agents to mimic BSYJF capsules. Placebo capsules are asked identical to BSYJF capsules in size, appearance, color, favor and weight.

11. CALCULATION OF SAMPLE SIZE (This section has been amended. Please refer to the information in "Amendment" section.)

The estimated measurement variability in BMD, muscle (lean mass of left thigh) and functional mobility is 0.72, 0.89 and 0.89, respectively. The minimum expected difference in BMD between BSYJF and placebo is defined to be 0.5. Therefore, a minimum sample size of 37 for BMD, and 55 for lean mass of left thigh and functional mobility, respectively. Each group will provide 90% statistical power at the significance level of 5% in the one-sided test.

For falls, we hypothesize that BSYJF group will lead to an annual 50% reduction, and the incidence of falls among community-dwelling elderly women is considered to 19% in the placebo group according to the previous study (Aoyagi and Ross et al., 1998; Kannus, 1999). A sample size of 110 (55 per

group) is based on a one-sided significance level of 5%, and a power of 90%. Assuming the drop-out rate is 20%, 70 patients per group will be recruited for the trial.

12. Measurements (This section has been amended. Please refer to the information in “Amendment” section.)

During the study, every effort should be made to keep subjects on the study schedule of procedures (Table 1). These measurements should be performed at baseline and 12, 24, and 36 months. The study window is ranging from -7 to 7 days.

12.1 BMD and Muscle Measurement

All the densitometry operators in the two sites should train specifically to ensure the following standard procedures. Left proximal femur (femoral neck and lean mass) in anteroposterior projection will be measured by DXA (DPX-L; Lunar, Madison, WI, USA). The same DXA apparatus should be used for a specific patient over the entire period. Monitoring of DXA scanner drift should be achieved by regular scanning of a calibration phantom.

12.2 TUG Test

TUG test, consisting of both walking speed and chair rise components, is designed to evaluate the functional mobility of the participants. The participants will be instructed to rise from a standard arm chair, walk 3 meters, turn around, walk back to the chair at the normal pace and sit down (Podsiadlo and Richardson, 1991). Higher scores represent a poorer degree of functional mobility (Appendix C).

Table 1 Schedule of Assessments

Assessment	Screening	Baseline	Treatment		
	Wk. −2-0	Wk.0	Mo. 12	Mo. 24	Mo. 36
General information					
Informed consent	√				
Medical/Medication history	√				
Physical exam (Height & Weight)	√		√	√	√

BMD screening	√				
Observations					
BMD and lean mass		√	√	√	√
TUG test		√	√	√	√
Falls		√	√	√	√
Bone biomarkers		√	√	√	√
Hematology		√	√	√	√
ECG		√	√	√	√
B-ultrasound		√	√	√	√
DDIPA		√	√	√	√
Adverse events			√	√	√
Others					
Drug dispensing		√	√	√	
Drug return			√	√	√
Combined disease and Medications	√		√	√	√

12.3 Falls Recording

A fall is defined as any event that led to an unplanned, unexpected contact with a supporting surface (Podsiadlo and Richardson, 1991). Falls resulting from pathologic fracture, major trauma or facial trauma, and/or unavoidable hazards such as transient ischemic attacks, chair collapsing or cerebrovascular accidents will be excluded. A faller is defined as the participants sustained at least one fall or more. Information about new falls while on study will be documented using form, included the date of fall and related-injury. The number of falls is accumulated. A copy of radiograph confirming the fracture will be obtained.

12.4 Laboratory Assessments

All screening and on-study samples will be processed and sent to the laboratory. The laboratory will be responsible for performing the assays. Biochemical examination (complete blood count, renal function, liver function and 25-OH-vitamin D), blood pressure and observation of abnormal symptoms/signs based on standardized patient report forms performs at

baseline and 12, 24, and 36 months (Table 1). Serum osteocalcin or urine deoxypyridinoline is as a marker predicting hip fracture in elderly women (Szulc and Chapuy et al., 1996). Serum 25-OH-vitamin D, osteocalcin, 17β-estradiol and urine deoxypyridinoline should be obtained in the morning after an overnight fast and be delivered to the laboratory for measurements using commercial kits.

12.5 Uterus Endometrial Thickness Monitoring

We will monitor uterus endometrial thickness using transvaginal ultrasound (Siemens Medical Solutions Systems, Issaquah, WA, USA). Endometrial thickness will be measured at the thickest portion of the endometrium and be included both endometrial layers. The sensitivity of detecting endometrial abnormalities is 92% and for detecting cancer is 96% when an endometrial thickness of 5 mm is used as the upper limit of the normal value (Smith-Bindman and Kerlikowske et al., 1998). During each clinical visit, abnormalities should be evaluated and documented, and follow-up medical care should be provided as needed.

13. SAFETY DATA REPORTING

13.1 Adverse Events Definitions

An adverse event is defined as “any untoward medical occurrence in a patient or clinical investigation subject administered a pharmaceutical product and that does not necessarily have a causal relationship with this treatment.”

This definition of adverse events is broadened in this study to include any such occurrence (eg, sign or symptom) or worsening of a pre-existing medical condition from the time that a subject has signed informed consent to the time of initiation of BSYJF or placebo. Worsening indicates that the pre-existing medical condition (*e.g.* migraine headaches and gout) has increased in severity, frequency, or duration of the condition or an association with significantly worse outcomes.

The investigator is responsible for reviewing laboratory test results and determining whether an abnormal value in an individual study subject represents a change from values before the study. In addition, interventions for pretreatment conditions or medical procedures that were planned before study enrollment are not considered adverse events. Abnormal laboratory findings without clinical significance (based on the investigator's judgment) should not

be recorded as adverse events; however, laboratory value changes requiring therapy or adjustment in prior therapy are considered adverse events.

13.2 Serious Adverse Events

A SAE is defined as an adverse event that:

- is life threatening or fatal;
- is a congenital defect;
- requires hospitalization;
- results in persistent or significant disability/incapacity;
- major bleeding (*e.g.* gastro-intestinal or intracerebral);
- end-stage renal disease requiring renal replacement therapy;
- any other major health conditions/events.

A hospitalization meeting the regulatory definition for “serious” is any inpatient hospital admission that includes a minimum of an overnight stay in a health care facility. Any adverse event that does not meet one of the definitions of serious (eg, emergency room visit, or requires urgent investigation) may be considered by the investigator to meet the “other significant medical hazard” criterion for classification as a SAE.

13.3 Adverse Event Collection and Reporting

Any adverse events occurring after the first dose of study medication will be recorded in the subjects’ records and on the CRFs. Information about the occurrence of any adverse events will be sought at all scheduled visits. The investigator is responsible for ensuring that all adverse events observed by the investigator or reported by subjects if SAE occurs. The SAE is reported within 24 hours to the coordinator by completing a Serious Adverse Event Form (Appendix D). In addition, the investigator should ensure that all regulatory requirements are completed.

The following adverse event attributes must be assigned by investigator: diagnosis, signs or syndrome(s) (if known); dates of onset; severity; assessment of relatedness to BSYJF/placebo and action taken. The investigator may be asked to provide follow-up information, discharge summaries, and extracts from medical records or CRFs.

A subject may voluntarily withdraw from treatment due to what she perceives as an intolerable adverse event. If either of these situations arises, the subject

should be strongly encouraged to undergo an end-of-study assessment and be under medical supervision until symptoms cease or the condition becomes stable. Documentation of all such SAEs will be retained in medical records.

13.4 Safety Monitoring

Monitoring visits will occur on at least 2 occasions in the first year and on at least one occasion each year thereafter. Sites will provide 3-monthly progress reports with information on participant, adverse events, and any protocol deviations. PI will review the reports from the site monitoring visits and the site progress reports every year.

The report will include:

- A list and summarization of adverse events by age and other characteristics
- Whether adverse event rates are consistent with pre-study assumptions;
- Reason for dropouts;
- Whether all participants met entry criteria;
- Whether continuation of the study is justified on the basis that additional data are needed to accomplish the stated aims of the study;
- Conditions whereby the study might be terminated prematurely.

14. DROPOUT

If any investigator encounters a participant dropout, such as moving away from proximity of the site, incompatible with the intervention (withdrawal), and discontinued the intervention over 3 months. The decision of participant dropout will be reported to the required PI.

Participants will continue to be followed with their permissions if the study intervention is discontinued at the site. These participants will be followed up annually for 36 months for clinical outcomes and adverse events of (1) death from any cause; (2) hospitalization event. We will evaluate compliance by counting the residual at each visit (practical dose/dose application × 100%). Good compliance means the drug actually taken equals 80% to 120 % of the required dosage.

15. ENDPOINTS (This section has been amended. Please refer to the information in “Amendment” section.)

15.1 Primary Endpoints

(1) Change from month baseline at month 36 in femoral neck BMD;
(2) Change from month baseline at month 36 in lean mass of left thigh;
(3) Change from month baseline at month 36 in TUG test;
(4) The number of falls during the 36 months.

15.2 Secondary Endpoints

(1) Change from month baseline at month 36 in osteocalcin;
(2) Change from month baseline at month 36 in deoxypyridinoline;
(3) Changes from baseline at month 36 in estradiol;
(4) Change from month baseline at month 36 in endometrial thickness;
(5) Nature, frequency and severity of adverse events and their relationship to treatment.

We will also assess vital signs, laboratory values and electrocardiographic variables during the 36-month follow-up.

16. STATISTICAL CONSIDERATIONS (This section has been amended. Please refer to the information in “Amendment” section.)

16.1 Data Collection Forms

Patient information collected for the trial is recorded on the appropriate CRFs. All completed CRFs must be submitted to the sponsor at the end of month 36.

16.2 Data Management

After confirming the correct database established by PI, the pre-defined analysis plan in the study should be finalized before database lock and unblinding. Under the supervision of the trial project manager, a data management system will be administered. The data management system for the trial is in-built. The inspector will monitor enrollment, loss to follow up, adherence and satisfaction with intervention, as well as adverse events. These regulations that ensure complete patient and data safety and confidentiality will be instituted and documented meticulously.

16.3 Statistical Analysis

All the analyses will use randomly assigned patients under ITT principle. For patients missing data, the LOCF approach use to analyze the longitudinal data. Repeated-measures ANOVA analysis should be carried out between two groups and four times (baseline, 12, 24 and 36 months). Within-subjects results will be used *t*-test on the percentage change from the baseline value. The

numbers of falls and adverse events will perform by chi-square test. All tests will be used two-sided and set at the 5% level.

17. QUALITY ASSURANCE

17.1 Training

The study will be conducted in accordance with GCP and with all relevant regulations. All research staff will be trained on the study protocol and procedure manuals before the start of participant recruitment. All required study information must be recorded on medical documents or CRFs. Study data derived from laboratories and subject follow-up visits, clinic attendance, and screening will all be collected with corrective actions executed as required throughout the duration of the study.

17.2 Diet and Exercise Control

During the entire trial, the subjects will be advised to follow the guideline for daily dietary intake and physical activities, such as intaking high-protein diet, avoiding alcohol abuse and smoking, and keeping active in daily physical activities. The questionnaire for daily dietary intake and physical activities (DDIPA; Appendix F) should be recorded at 12, 24, 36 months during 36-month clinical trial. Calcium and vitamin D supplement on their own should be prohibited in 36-month clinical trial.

17.3 Data Quality

The PI will inspect an on-site study that contain the study protocol, manual of procedures, informed consent form, IRB document, medication records, general trial correspondence and patient screening logs for reference. The PI is responsible for ensuring regular submission of the completed CRFs in monitoring and analysis. In the event of incomplete, incongruous or ambiguous data, the investigator and/or coordinator will be contacted for clarification. The PI is responsible for data accuracy, consistency and quality.

CRFs and biological samples will be labeled with a unique study identifier. Ten percent of CRFs will be duplicated and the copy will be used for double data entry. All quantitative data will be entered into a Microsoft Excel and audited for accuracy. Coded forms will be kept separately from the code list to maintain confidentiality information. All forms will be stored in locked file

cabinets or desks in a locked office.

All worksheets, documents and administrative records should be retained by the investigator. The investigator should refer to the associated procedures manual for further information regarding details of the procedures to be followed during the course of the trial.

17.4 Monitoring

The purpose of visits will be to ensure that the study is conducted according to the protocol and GCP guidelines. These quality control reviews will also inspect study records and source documents for specific verification of participant details, data quality, and completeness of intervention implementation.

Access to CRF, source documents, and other study files must be made available at two sites for monitoring and audit purposes at these monitoring visits during the course of the study and after the study. Any deviations will be documented.

17.5 Interim Analysis and Stopping Guidelines

An interim analysis will be performed at month 12. Although this is a 3 year study, the primary analysis will occur after all subjects have had the opportunity to complete the month 12 study visit. In order to maintain the blind, study staff at the Office of Scientific Research and Teaching who have direct contact with the sites will have access to results from the interim and primary analyses only at aggregate level until all subjects have completed the study and the treatment assignments have been unblinded. The month 36 data will be analyzed separately at the end of the study.

The study staff at the Office of Scientific Research and Teaching will review the safety and efficacy data for BSYJF/placebo. If at any time there are safety concerns, the study staff will communicate the concerns with a coordinator. The start date will depend on the subject’s accrual enrollment.

18. PARTICIPANTS' RIGHTS AND CONFIDENTIALITY

18.1 Institutional Review Board

This protocol, ICF and any subsequent modifications will be reviewed and approved by IRB for written approval, which is responsible for oversight of

the study. The investigator should notify the IRB of deviations from the protocol or SAE occurring at the site. The study may be discontinued at any time by the IRB as part of their duty to ensure that participants are protected.

18.2 Subject Confidentiality

CRFs will be stored in locked cabinets at participating clinics and will be accessed only by permitted study staff and the monitor from the Office of Scientific Research and Teaching. Names and other easily recognizable identifiers will be removed from CRFs prior to data entry and analysis. Instead, identification numbers will be used so that data and specimens may be linked; these are not meaningful to casual observers without access to the original study logs. Any data, specimens, forms, reports, and recordings that leave the site will be identified only by the identification number to maintain confidentiality.

The study records will be available to regulatory agencies, such as IRB. All trial staff will be trained in procedures to minimize the potential breaches of confidentiality, including but not limited to, ensuring that all files are closed and no conversations about individual participants occur in public.

18.3 Conflict of Interests

The sponsor has established a policy regarding Conflict of Interests to be adhered to by investigators. The policy will provide rules to conduct the trial in an unbiased and informed manner that meets public standards.

19. PUBLICATION POLICY

To coordinate dissemination of data from this study, data will be reviewed for validity and subsequent publication. The investigators will develop a suitable policy protecting the rights regarding ownership of study materials and data. Briefly, manuscripts will be in the name of appliances; the supporters will be acknowledged. Each author should have participated sufficiently in the work to take public responsibility for appropriate portions of the content.

20. FUNDING

This trial is funded Ministry of Science & Technology of China (Project No.

96-906-09-05), and Shanghai Science & Technology Commission (Project No. 004319226).

21. REFERENCES

1. Aoyagi, K. and P. D. Ross, et al. (1998). "Falls among community-dwelling elderly in Japan." J Bone Miner Res **13** (9): 1468-74.
2. Chapuy, M. C. and M. E. Arlot, et al. (1992). "Vitamin D3 and calcium to prevent hip fractures in the elderly women." N Engl J Med **327** (23): 1637-42.
3. Cooper, C. and D. J. Barker (1995). "Risk factors for hip fracture." N Engl J Med **332** (12): 814-5.
4. Greenspan, S. L. and E. R. Myers, et al. (1994). "Fall severity and bone mineral density as risk factors for hip fracture in ambulatory elderly." JAMA **271** (2): 128-33.
5. Kannus, P. (1999). "Preventing osteoporosis, falls, and fractures among elderly people. Promotion of lifelong physical activity is essential." BMJ **318** (7178): 205-6.
6. Podsiadlo, D. and S. Richardson (1991). "The timed "Up & Go": a test of basic functional mobility for frail elderly persons." J Am Geriatr Soc **39** (2): 142-8.
7. Shen, P. and Y. Xu, et al. (1997). "Effects of Migu capsule and Vitamin D3 on bone mass and bone strength in glucocorticoid-induced osteoporosis model." Chin J Osteoporosis(03): 9-11+8.
8. Smith-Bindman, R. and K. Kerlikowske, et al. (1998). "Endovaginal ultrasound to exclude endometrial cancer and other endometrial abnormalities." JAMA **280** (17): 1510-7.
9. Song, X. and P. Shen, et al. (1998). "Effect of kidney tonifying Migu tablet on osteoporosis rats bone and endocrine." Chin J Integr Trad West Med **0** (S1): 277-279.
10. Song, X. and Y. Shi, et al. (1995). "Experimental study of Chinese kidney-nourishing herb on postmenopausal osteoporosis." J Shanghai Railway Med Colloge. **9** (3): 141-145.
11. Song, X. and Y. Shi, et al. (1996). "Study of Chinese kidney-nourishing herb on experimental postmenopausal osteoporosis and mechanism." Chinese Journal of Traditional Medical Traumatology & Orthopedics **4** (3): 5-8.
12. Song, X. and Y. Shi (1997). " Chinese kidney-nourishing herb on

osteoporotic rat skeleton." China J Orthop Traumatol. **10** (5): 13-14.

13. Song, X. and Z. Song, et al. (1997). "Clinical Observation on Prevention and Treatment of Postmenopausal Osteoporosis with Chinese kidney-nourishing herb." The Practical Journal of Intergrating Chinese with Mordern Medicine **10** (9): 892.
14. Szulc, P. and M. C. Chapuy, et al. (1996). "Serum undercarboxylated osteocalcin is a marker of the risk of hip fracture: a three year follow-up study." Bone **18** (5): 487-8.
15. Zhang, G. and W. Shi, et al. (1999). "Evaluation of Bushen Yijing Fang in delaying the aging of male rats and its mechanism." The Journal of Traditional Chinese Orthopedics and Traumatology **11** (09): 3-5+62.
16. Zhang, G. and W. Shi, et al. (1999). "Experimental study of Chinese kidney-nourishing herb delay bone loss age-related male femoral cortical bone and cancellous bone on rats." Chin J Osteoporosis. **5** (3): 51-54.
17. Zuckerman, J. D. (1996). "Hip fracture." N Engl J Med **334** (23): 1519-25.

22. APPENDICES

Appendix A. Human Ethics Approval

Institutional Review Board Approval

Ethics number: SZYGSLL-1999-002

The basic information of the submitting project:

- Project Name: "The effects and safety of Bushen Yijing Fang reduces hip fractures risk factors in late postmenopausal women with osteopenia: A long-term follow-up, randomized, double-blind and placebo-controlled trial"
- Applicants: Shi Yinyu; Zhang Ge; Zheng Yuxin
- Type of registration: research project
- Submission lists: study protocol, informed consent form, case report form

Members of the Ethics Committee (by names)

Name	Gender	Title	Institute	Remarks
Shi Guantong	male	chief physician	Shanghai Shuguang Hospital	chairman
Shen Peizhi	female	associate researcher	Shanghai Academy of Traditional Chinese Medicine	
Wang Hongfu	male	chief physician	Shanghai Institute of Radiation Medicine	
Wang Shiqin	female	senior teacher	Education Bureau of Xuhui District	
Xu Rongxi	male	deputy chief physician	Shanghai Shuguang Hospital	

Review result:

The Ethics Committee has reviewed and agreed the project program, which meets the ethical requirements and national regulations.

Date: August 23 1999

Chairman signature: Shi Guantong

Ethics Committee of Orthopaedics and Traumatology Institute with Shanghai Academy of Traditional Chinese Medicine

Appendix B: Patient Information Sheet and Informed Consent Form

Ver1.0; August/1999
(Please keep the original of this form in the investigator's file and give one copy to the participant)

Investigator: **Site:**

Study Title: "The effects and safety of Bushen Yijing Fang reduces hip fractures risk factors in late postmenopausal women with osteopenia: A long-term follow-up, randomized, double-blind and placebo-controlled trial"

Coordinating Centre: Shuguang Hospital

Sources of Funding: Funding by China Science and Technology Project (96-906-09-05) and Shanghai Science and Technology Commission (004319226)

Introduction

You are invited to participate in a study for late postmenopausal women with osteopenia at least 10 years after natural menopause, aged from 55 to 69 years, T-score between −2.5 and −2 SD at femoral neck at least 12 months. These conditions increase your risk of the fracture. This study tests whether BSYJF reducing hip fracture risk factors than placebo.

Your participation is entirely voluntary. To help you make your decision, please read this information sheet. You are free to discuss the contents of this document with members of your family or your physician. You may take as much time as you like to consider whether or not to take part in the study. If you choose not to take part, your current or future care will not be affected. If you agree to take part, you are free to withdraw from the study at any time without penalty or loss of benefits.

Once you understand what is involved in the study and you wish to participate, you, along with your study doctor, will be asked to sign the consent form. If

you have questions at any time during the research study, you should feel free to ask him/her and obtain answers to your questions. You are not giving up any of your rights by volunteering for this trial or by signing this consent form.

Study Purpose

This study is to research the long-term effects of BSYJF on bone-dependent and bone-independent hip fractures risk factors in late postmenopausal women in osteopenic patients. A total of 140 participants will be selected from two sites in Shanghai to participate for 36 months.

You will be randomly assigned to one of the two groups: (1) BSYJF + calcium, or (2) placebo + calcium. The treatments given to you in both the groups will remain the same. However, participants in BSYJF group will receive more active interventions. There is a 50% chance of being assigned to either of the groups. That’s to say, the decision is made randomly, and each person in the group is decided all by chance.

During the Study

If you agree to participate, you will be seen at the beginning of the study on 3 occasions maximally. The first 2 visits will be to see if you are eligible for the study and the third visit is assigning you to your treatment group. For the first visit, you will have a brief medical history taken. If you still qualify for the study, you will need to come back within 1 week after fasting overnight for the second visit to do hip BMD measurement, transvaginal ultrasound and blood test to check your T-value, endometrial thickness, and kidney and liver function, etc. The amount of blood taken will be approximately 2 teaspoons (10 ml). A urine test for deoxypyrdinoline test will also be done. Depending on the results of the previous tests, you will be informed whether or not you qualify to continue on with the study for the third visit. If you qualify, we will do a full history and physical exam, and other questionnaires about your care. Afterwards, we will provide you your group assignment.

Follow-up Visits

The expected time that you will be followed-up for this trial is 36 months. At every 12-monthly visit, you will be called back; we will obtain the number of falls, hip BMD measurement, transvaginal ultrasound, laboratory tests, and TUG exam to monitor hip fracture risk factors.

Risks

This study will not cause you any special harm or discomfort more than your existing care for osteopenia. When blood is drawn for lab tests during the study, there is a possibility of bruising, discomfort from the needle puncture, and infection. Besides, there are some adverse reactions, such as stomach discomfort, and/or nausea. You may not participate if you BMD become worse during the course of the study, you should notify the investigator of this fact immediately.

Benefits

You may become more aware of your condition and treatments with increased clinic visits during follow-up treatment. Information gathered from this study may be helpful to the future management of osteoporosis and hip fracture risk factors. However, we do not know long-term effects of BSYJF, so you may receive no direct benefit from participating in this study.

Participation in this study will be at no cost to you. There will be no monetary compensation for your participation.

Alternative Procedures

Different treatments for late postmenopausal women with osteopenic patients have been assessed on an individual basis. Some physicians use calcium for osteopenia follow-up, which is similar to our study. Some ones use exercise or physical activity for falls. However, whether these interventions have long-term efficacy for hip fracture risk factors is not known. This study aims to bring bone-dependent and bone-independent together into one comprehensive care package to modify the risk factors of hip fractures.

Confidentiality

The research staff will collect information related to your health if your participation in this study. Your collected information will be stored locally and will be kept in a secure location with access limited to authorized personnel only. We will store and process your information with electronic data processing systems. In the electronic database, your information will be identified only with a code number. At the end of the study, all personal identifiers will be destroyed. Samples for long-term storage will only be identified with a code number and will be stored at the laboratory. The stored

blood samples will be accessed only by the research team for later analyses.

By signing this informed consent form, you are agreeing to allow the study monitors, government regulatory agencies, and IRB to examine your medical records. Your name will be kept confidential to the extent allowed by law, and you will not be identified personally in any presentations or reports with this research. When the results of the study are published, your identity will not be revealed.

Withdrawal

Whether or not to participate in this study is completely voluntary. You may withdraw from the study at any time for any reason. If you decide to withdraw from the study before the finish, you will be asked to provide the reason for withdrawal, but you have the option not to provide the reason. In addition, there will be no penalty or loss of benefits to you if you decide not to participate or decide to withdraw from the study. Your participation can also be stopped by your investigator or the study sponsor for the following reasons:

- Any serious side effects appears;
- If you do not comply with the requirements of the study;
- If your investigator or the sponsor has the opinion that it would be in your best interest to withdraw from the study.

Enquiries

If you have any questions about the research, develop a research-related problem, or note a change in your condition, you should contact the Investigator [Name, address and telephone number].

Should you have any questions regarding your rights as a research participant, you may contact the IRB of [Name, address and telephone number].

Consent Form

Ver1.0; August/1999

(Please keep the original of this form in the investigator's file and give one copy to the participant)

Study Title: The effects and safety of Bushen Yijing Fang reduces hip fractures risk factors in late postmenopausal women with osteopenia: A long-term follow-up, randomized, double-blind and placebo-controlled trial

Participant: Mr/Ms ___________ Years______/____/___;___

Name Birth Date (yyyy/mm/dd); Age

Please read the following points before putting your signature:	Place[√]
1. I confirm that I have read and understood the participant information sheet for the study and have had the opportunity to ask questions.	[ ]
2. I understand that my take part in the study is voluntary and that I am free to withdraw at any time, without giving any reason, without my medical care or legal rights being affected.	[ ]
3. I understand that the Sponsor of the study, others working on the Sponsor's behalf, the Ethics Committee, and the regulatory authorities will not need my permission to look at my health records, both for the current study and any further research that may be conducted in relation to it, even if I stop participating in the study. I understand that my identity will not be revealed in any information released to third parties or published.	[ ]
4. I agree not to restrict the use of any of my information or results that arise from this study provided such a use is only for scientific purpose.	[ ]
5. I have been given a copy of the information sheet and consent form to keep. By signing this form I have not given up my legal rights.	[ ]

Signature of Subject **Date**:__/__/__

Signature of Investigator **Date**:__/__/__

Signature of the Witness (or Legal Representative) **Date**:__/__/__

Physician Interview Information Sheet and Consent Form

Ver1.0; August/1999

(Please keep the original of this form in the investigator's file and give one copy to the investigator)

Study Title: The effects and safety of Bushen Yijing Fang reduces hip fractures risk factors in late postmenopausal women with osteopenia: A long-term follow-up, randomized, double-blind and placebo-controlled trial

Principal Investigator: Dr. Shi Yinyu
Site: [Name]
Sponsor: Shuguang Hospital

<u>Introduction</u>

You are a study physician in this trial, which is to evaluate long-term effects of BSYJF on hip fracture risk factors in late postmenopausal with osteopenia. We are inviting your participation in interviews throughout the trial. Your feedback will help improve the intervention to modify hip fracture risk factors.

This form is designed to tell you everything you need to understand before you decide to consent to be or not to be in the study. It is entirely your choice. If you decide to take part, you can change your mind later on and withdraw from the trial. The decision to join or not join the research study will not cause you to lose any benefits.

<u>Procedures</u>

If you agree to participate, you will be taking part in 6 interviews maximally over the course of the trial. Each interview will last approximately 15 minutes and will be conducted at your office or another mutually agreed upon location. During the baseline interview, the questions will be about your present practice in hip fracture risk factors and the challenges and successes you have in patient management; and also your views on the feasibility of the intervention. During the 3 follow-up interviews, you will be asked about the intervention's progress

and the effects on patient, and your views on its sustainability. Your privacy will be protected safely and your name will not be used in the recording.

Benefits

Your feedback from the interview will be used to improve evidence of BSYJF on hip fracture risk factors in late postmenopausal with osteopenia. After the trial, the goal is that the tested BSYJF can be scaled-up for use in other clinics to improve falls and hip fracture.

Risks

There are no foreseeable risks or discomforts associated with this study. You may stop the study at any time.

Compensation

Your participation is completely voluntary. No material compensation will be provided for your participation. Your help with this study is greatly appreciated.

Confidentiality

A study number rather than your name will be used on data collected. The code that links the number to your name will be kept in a secure place. All research records will be kept in a locked cabinet. Your name and other facts that might identify you will not appear in study results. Any shared data will not include any identifiable information. People other than those doing the study may look at study records: agencies and committees that make rules and policy about how research is done have the right to review these records.

Withdrawal of Participation

Participating in the interviews is voluntary, and you may leave the study at any time without penalty. This decision will not affect in any way your current or future care or any other benefits to which you are otherwise entitled. You may also refuse to answer any questions that you do not want to during the interview.

Enquiries

If you have questions about your rights as a research or if you have

questions, concerns or complaints about the research, you may contact Dr. Shi Yinyu: Address: 185 Pu'an Road, Shanghai; Phone: 53821650.

Consent

Do not sign this consent form unless you have had a chance to ask questions and get answers that make sense to you. Nothing in this form can make you give up any legal rights. By signing this form you will not give up any legal rights.

You will receive a copy of this consent form to keep. Please sign below if you agree to participate in this study.

Name of Physician-Participant

Signature of Physician-Participant **Date**:____/___/___

Interviewer **Date**:____/____/___

Appendix C. Falls Recording Form

Record No. of each fall	Date (yy-mm-dd)	Perceived Causes	Body site to touch ground	Injury	Fracture
1		☐ Balance/gait impairment<br>☐ Dizziness/vertigo<br>☐ Accident<br>☐ Others	☐ Wrist<br>☐ Shoulder<br>☐ Hip<br>☐ Others	☐ No injury<br>☐ Untreated injury<br>☐ Treated injury	☐ No fracture<br>☐ Vertebral<br>☐ Hip<br>☐ Others
2		☐ Balance/gait impairment<br>☐ Dizziness/vertigo<br>☐ Accident<br>☐ Others	☐ Wrist<br>☐ Shoulder<br>☐ Hip<br>☐ Others	☐ No injury<br>☐ Untreated injury<br>☐ Treated injury	☐ No fracture<br>☐ Vertebral<br>☐ Hip<br>☐ Others
3		☐ Balance/gait impairment<br>☐ Dizziness/vertigo<br>☐ Accident<br>☐ Others	☐ Wrist<br>☐ Shoulder<br>☐ Hip<br>☐ Others	☐ No injury<br>☐ Untreated injury<br>☐ Treated injury	☐ No fracture<br>☐ Vertebral<br>☐ Hip<br>☐ Others
4		☐ Balance/gait impairment<br>☐ Dizziness/vertigo<br>☐ Accident<br>☐ Others	☐ Wrist<br>☐ Shoulder<br>☐ Hip<br>☐ Others	☐ No injury<br>☐ Untreated injury<br>☐ Treated injury	☐ No fracture<br>☐ Vertebral<br>☐ Hip<br>☐ Others
5		☐ Balance/gait impairment<br>☐ Dizziness/vertigo<br>☐ Accident<br>☐ Others	☐ Wrist<br>☐ Shoulder<br>☐ Hip<br>☐ Others	☐ No injury<br>☐ Untreated injury<br>☐ Treated injury	☐ No fracture<br>☐ Vertebral<br>☐ Hip<br>☐ Others

Note: A fall is defined as any event that led to an unplanned, unexpected contact with a supporting surface.

Appendix D. Timed Up and Go (TUG) test

The TUG test is designed to evaluate the functional mobility of the participants. Subjects observe and time from the instant they rising from an armchair, walking to a mark 3 meters away, turning around and returning to sit back in the chair at a regular pace. Subjects wear their usual footwear and allow using the arms of the chair to get up. Subjects begin the test on the word, “go” and are instructed to “walk”. Each subject performs TUG test 3 times and the fastest of these tests is finally recorded in seconds. A practice trial uses to test each subject understanding. Same chair is used for re-testing during the trial.

Appendix E. Adverse Event Severity Scoring System

Grade	Common Terminology Criteria
1 Mild	Aware of sign (s) or symptom (s), but easily to tolerate
2 Moderate	Discomfort enough to cause interference with usual activity
3 Severe	Incapacitating with inability to work or do normal activity
4 Life threatening	Refers to an event in which the participant was, in the view of the investigator, at risk of death at the time of the event.
5 Fatal	

Appendix F. Questionnaire for Daily Dietary Intake and Physical Activities

Name:_____________ Date:_______________

Part I. Daily dietary intake

1. The choice of your staple food is:
□ fine rice and flour with coarse grains
□fine rice and flour, occasionally coarse grains
□ only fine rice and flour

2. Do you often eat fresh vegetables daily?
□ rarely
□ a little, less than 400 grams
□ 400 grams or more

3. Do you often eat fresh fruit daily?
□ rarely
□ a little, less than 100 grams
□ 100 grams or more

4. Do you often eat aquatic products weekly?
□ occasionally or once
□ 2-3 times on average
□ 3 times or more

5. Do you often eat eggs weekly?
□ 1-3
□ 4-6
□ More than 7

6. Do you drink milk daily?
□ rarely
□ a little, less than 200 grams
□ 200 grams or more

7. Do you often eat soy products daily?
□ rarely
□ a little, less than 50 grams
□ 50 grams or more

Part II. Daily movement

1. How often do you have a (an) fitness/exercise weekly?
□ 1 time or none
□ 2 times
□ 3 times
□ 4 times
□ 5 times or more

2. How long you will take to do an exercise?
□ 30 min or less
□ 30-60 min
□ 60-90 min
□ 90-120 min
□ 120 min or more

3. What sports do you often attend? [Multiple choice]
□ swimming, running or walking
□ climbing or other outdoor sports
□ basketball, badminton or other ball games
□ aerobics or dance
□ Tai Chi, Qigong, Yoga
□ others

23. AMENDMENTS 1:

Protocol Title: The effects and safety of Bushen Yijing Fang (BSYJF) reduces hip fractures risk factors in late postmenopausal women with osteopenia: A long-term follow-up, randomized, double-blind and placebo-controlled trial

Major amendment: Falls as Primary Endpoint
Amendment Date: 10 December 1999
Version number: Ver1.1

Rationale:

Hip fractures usually occur from a fall in the elderly women. Therefore, identifying why the fall occurs and implementing treatments, is a key to reducing the occurrence of hip fracture. So falls constitutes strongly the target outcomes of the trial. In line with the updated recommendation by ethnic committee, numbers of falls is selected as the only primary endpoint.

Description of Changes:

Endpoints, simple size, statistical considerations were updated to reflect the change in this study design.

Section: 2. Protocol Synopsis

**Protocol name**	**The effects and safety of Bushen Yijing Fang (BSYJF) reduces hip fractures risk factors in late postmenopausal women with osteopenia: A long-term follow-up, randomized, double-blind and placebo-controlled trial**
Sponsor	Shanghai Shuguang Hospital
Indication	Late postmenopausal women with osteopenia
Purpose	To evaluate long-term efficacy of BSYJF on hip fracture risk factors in late postmenopausal women with osteopenia.
Design	Randomized, 36-month, double-blind and placebo-controlled trial
Simple size	140 subjects (70 per group)
Setting	Two sites
Intervention time	From 2000-01-01 To 2004-12-31
Eligibility criteria	Inclusion:<br>1. Women with at least 10 years natural menopause, and aged from 55 to 69 years;<br>2. Osteopenia at least 12 months (T-score between −2.5 and −2 SD at femoral neck).<br><br>Exclusion:<br>1. Who had been diagnosed as having a neurological or musculoskeletal disorder, or coexisting chronic diseases;<br>2. Who have taken estrogen and calcitonin, fluoride, bisphosphonates, or adrenocortical hormone within one year;<br>3. Who had been took ≥ 4 prescription medications;<br>4. has environmental hazards for falls or tripping;<br>5. has impairment in gait;<br>6. has postural hypotension: drop in systolic blood pressure ≥20mmHg or to <90mmHg on standing;<br>7. has impairment in transfer skills or balance;<br>8. has impairment in leg or arm muscle strength or range of motion (hip, ankle, knee, shoulder, hand, elbow) ;<br>9. has ALT or AST levels greater than 50% of upper normal limit; serum creatinine levels greater than 133μmmol/l or

	1.5 mg/dl.
Drug administration	300mg element calcium daily with either BSYJF capsules (3 capsules per time, 3 times per day) or placebo.
Study period	36-month
**Endpoints**	**Endpoints are to be evaluated for BSYJF versus placebo.**<br>**<u>Primary endpoint</u>:**<br>**1. Number of falls at month 36.**<br><br>**<u>Secondary endpoints</u>:**<br>**1. Change from month baseline at month 36 in femoral neck BMD;**<br>**2. Change from month baseline at month 36 in lean mass of left thigh;**<br>**3. Change from month baseline at month 36 in TUG test;**<br>4. Change from month baseline at month 36 in biomarkers of bone turnover (osteocalcin and deoxypyridinoline);<br>5. Change from month baseline at month 36 in estradiol;<br>6. Nature, frequency and severity of adverse events and their relationship to treatment.
**Statistical considerations**	**All the analyses will use randomly assigned patients under ITT principle. For patients missing data after the baseline, the LOCF approach use to analyze the longitudinal data.**<br>**The numbers of falls and adverse events will perform by chi-square test. Kaplan–Meier analysis will be calculated in order to demonstrate differences in number of fallers over time. The time to first fall will be analyzed using a Cox regression analysis providing a 95% confidence interval.**<br>**Repeated-measures ANOVA analysis should be carried out between two groups and four times (baseline, 12, 24 and 36 months). Within-subjects results will be used *t*-test on the percentage change from the baseline value. All tests will be used two-sided and set at the 5% level.**

Section: 11. Calculation of sample size

Delete:

The estimated measurement variability in BMD, muscle (lean mass of left thigh) and functional mobility is 0.72, 0.89 and 0.89, respectively. The minimum expected difference in BMD between BSYJF and placebo is defined to be 0.5. Therefore, a minimum sample size of 37 for BMD, and 55 for lean mass of left thigh and functional mobility, respectively, in each group will provide 90% statistical power at the significance level of 5% in the one-sided test.

Section: 12. Table 1 Schedule of Assessments (updated)

Assessment	Screening	Baseline	Treatment		
	Wk. −2-0	Wk.0	Mo. 12	Mo. 24	Mo. 36
General information					
Informed consent	√				
Medical/Medication history	√				
Physical exam (Height & Weight)	√		√	√	√
BMD screening	√				
Observations					
Falls		√	√	√	√
BMD and lean mass		√	√	√	√
TUG test		√	√	√	√
Bone biomarkers		√	√	√	√
Hematology		√	√	√	√
ECG		√	√	√	√
B-ultrasound		√	√	√	√
DDIPA		√	√	√	√
Adverse events			√	√	√
Others					
Drug dispensing		√	√	√	
Drug return			√	√	√

Combined disease and Medications	√		√	√	√

Section: 12.3 Falls Recoding

Replace:
A fall is defined as any event that led to an unplanned, unexpected contact with a supporting surface. Falls resulting from pathologic fracture, major trauma or facial trauma, and/or unavoidable hazards such as transient ischemic attacks, chair collapsing or cerebrovascular accidents will be excluded. A faller is defined as the participants sustained at least one fall or more. Information about new falls while on study will be documented using form, included the date of fall and related-injury. The number of falls is accumulated. A copy of radiograph confirming the fracture will be obtained.

With:
A fall is defined as any event that led to an unplanned, unexpected contact with a supporting surface. Falls resulting from pathologic fracture, major trauma or facial trauma, and/or unavoidable hazards such as transient ischemic attacks, chair collapsing or cerebrovascular accidents will be excluded. A faller is defined as the participants sustained at least one fall or more. Information about new falls while on study will be documented using form, included the date of fall and related-injury. The number of falls is accumulated. **The cumulative incidence of fallers will be calculated as the total number of participants with first fall at each time point divided by the number of participants at risk.** A copy of radiograph confirming the fracture will be obtained.

Section: 15.1 Primary Endpoint

Replace:
(1) Change from month baseline at month 36 in femoral neck BMD;
(2) Change from month baseline at month 36 in lean mass of left thigh;
(3) Change from month baseline at month 36 in TUG test;
(4) The number of falls during the 36 months.

With:
Primary endpoint:

Primary endpoint is the number of falls at 36 months.

Section: 15.2 Secondary Endpoints

Replace:
(1) Change from month baseline at month 36 in osteocalcin;
(2) Change from month baseline at month 36 in deoxypyridinoline;
(3) Changes from baseline at month 36 in estradiol;
(4) Change from month baseline at month 36 in endometrial thickness;
(5) Nature, frequency and severity of adverse events and their relationship to treatment.

With:
<u>Secondary endpoints</u>:
(1) Change from month baseline at month 36 in femoral neck BMD;
(2) Change from month baseline at month 36 in lean mass of left thigh;
(3) Change from month baseline at month 36 in TUG test;
(4) Change from month baseline at month 36 in osteocalcin;
(5) Change from month baseline at month 36 in deoxypyridinoline;
(6) Changes from baseline at month 36 in estradiol;
(7) Change from month baseline at month 36 in endometrial thickness;
(8) Nature, frequency and severity of adverse events and their relationship to treatment.

Section: 16.3 Statistical Analysis

Replace:
All the analyses used randomly assigned patients under the ITT principle. For patients missing data after the baseline, the LOCF approach was used to analyze the longitudinal data. Repeated-measures ANOVA analysis was carried out between two groups and four times (baseline, 12, 24 and 36 months). Within-subjects results was used *t*-test on the percentage change from the baseline value. The numbers of falls and adverse events were performed by chi-square test. All tests will be used two-sided and set at the 5% level.

With:
All the analyses will use randomly assigned patients under ITT principle. For

patients missing data after the baseline, the LOCF approach use to analyze the longitudinal data.

The numbers of falls and adverse events will perform by chi-square test. Kaplan–Meier analysis will be calculated in order to demonstrate differences in number of fallers over time. The time to first fall will be analyzed using a Cox regression analysis providing a 95% confidence interval.

Repeated-measures ANOVA analysis should be carried out between two groups and four times (baseline, 12, 24 and 36 months). Within-subjects results will be used *t*-test on the percentage change from the baseline value. All tests will be used two-sided and set at the 5% level.

24. AMENDMENTS 2:

Protocol Title: The effects and safety of Bushen Yijing Fang (BSYJF) reduces hip fractures risk factors in late postmenopausal women with osteopenia: A long-term follow-up, randomized, double-blind and placebo-controlled trial

Major Amendment: 10 Years Extension Phase
Amendment Date: 10 January 2002
Version number: Ver1.2

Rationale:

In the vast majority of cases, a hip fracture is a fragility fracture due to a fall or minor trauma in someone with osteoporotic bone. Risk factors of hip fractures observed are expected to be of long duration. However, BSYJF treatment was designed initially 36 months. Instead, a 10 year extension phase is added to explore the effect of transitioning from 36 months of treatment. The main objective of this extension phase is to evaluate if latent effects of BSYJF that might be lower fall rate and even hip fracture rate. In other words, 10 years extension follow-up of treatment cessation can be studied and is an accepted clinical approach.

Introducing a 10 years extension follow-up phase:

Active intervention will take place over 3 years until the end of 2004. After the trial, the recruited participants will be followed up in the 3rd, 6th, and 10th year to assess the latent effects of BSYJF interventions on fall and hip fracture incidence rate in late postmenopausal women.

Description of Changes:

Study purpose, design, excluded criteria, endpoints, statistical analysis and the schedule of assessments were updated to reflect this change in study design.

Additionally, the study staff will have access to unblinded safety and efficacy data to assess the benefit/risk profile while the study is ongoing in this extension phase.

Section: 2. Protocol Synopsis

**Protocol name**	**The effects and safety of Bushen Yijing Fang (BSYJF) reduces hip fractures risk factors in late postmenopausal women with osteopenia: A long-term follow-up, randomized, double-blind and placebo-controlled trial**
Sponsor	Shanghai Shuguang Hospital
Indication	Late postmenopausal women with osteopenia
Purpose	To evaluate long-term efficacy of BSYJF on hip fracture risk factors in late postmenopausal women with osteopenia.
**Design**	**Randomized, 36-month, double-blind and placebo-controlled trial with 10 years extension follow-up**
Simple size	140 subjects (70 per group)
Setting	Two sites
**Intervention time**	**From 2000-01-01 To 2004-12-31**
**Eligibility criteria**	<u>Inclusion:</u><br>1. Women with at least 10 years after natural menopause, and aged from 55 to 69 years;<br>2. Osteopenia at least 12 months (T-score between −2.5 and −2 SD at femoral neck).<br><br><u>Exclusion:</u><br>1. Who had been diagnosed as having a neurological or musculoskeletal disorder, or coexisting chronic diseases;<br>2. Who have taken estrogen and calcitonin, fluoride, bisphosphonates, or adrenocortical hormone within one year;<br>3. Who had been took ≥ 4 prescription medications;<br>4. has environmental hazards for falls or tripping;<br>5. has impairment in gait;<br>6. has postural hypotension: drop in systolic blood pressure ≥20mmHg or to <90mmHg on standing;<br>7. has impairment in transfer skills or balance;<br>8. has impairment in leg or arm muscle strength or range of motion (hip, ankle, knee, shoulder, hand, elbow) ;<br>9. has ALT or AST levels greater than 50% of upper normal limit; serum creatinine levels greater than 133μmmol/l or

	1.5 mg/dl.<br><br>**Additional exclusion criteria have been defined to protect subjects who have experienced a large bone loss or fragility fracture during the initial 36 month treatment phase of the study.**
Drug administration	300mg element calcium daily with either BSYJF capsules (3 capsules per time, 3 times per day) or placebo.
**Study period**	**36-month with 10 years extension follow-up**
**Endpoints**	Endpoints are to be evaluated for BSYJF versus placebo.<br>Primary endpoint:<br>1. Number of falls at month 36.<br><br>Secondary endpoints:<br>1. Change from month baseline at month 36 in femoral neck BMD;<br>2. Change from month baseline at month 36 in lean mass of left thigh;<br>3. Change from month baseline at month 36 in TUG test;<br>4. Change from month baseline at month 36 in biomarkers of bone turnover (osteocalcin and deoxypyridinoline).<br>5. Change from month baseline at month 36 in endometrial thickness;<br>6. Change from month baseline at month 36 in estradiol;<br>7. Nature, frequency and severity of adverse events and their relationship to treatment.<br><br>**Additional endpoint:**<br>**8. Number of falls at the end of 10 years extension follow-up.**
**Statistical considerations**	All the analyses will use all randomly assigned patients under ITT principle. For patients missing data after the baseline, the LOCF approach use to analyze the longitudinal data. The number of falls at month 36 and adverse events will perform by chi-square test. Kaplan–Meier analysis will be calculated in order to demonstrate differences in number of fallers over time. The time to first fall will be analyzed using a Cox regression

	analysis providing a 95% confidence interval. Repeated-measures ANOVA analysis should be carried out between two groups and four times (baseline, 12, 24 and 36 months). Within-subjects results will be used *t*-test on the mean percentage change from the baseline value. All tests will be used two-sided and set at the 5% level.<br><br>**Additional analysis:**<br>**Number of falls at the end of 10 years extension follow-up will be performed by chi-square test.**

Section: 3.3 Rational

Add:

After the trial, the recruited participants will be followed up in the 3rd, 6th and 10th year to assess the latent efficacy of BSYJF interventions on number of falls in late postmenopausal women. The serum 25(OH)D levels will be also measured at those three time-points.

Section: 5.1 Study Design

Replace:

The study duration is from January 2000 to December 2004. Subjects will be randomly assigned to receive BSYJF (n=70) or placebo (n=70) with a 1:1 allocation ratio. The study will carry out at the two sites (Institute of Orthopaedics and Traumatology, Shanghai Academy of TCM; and Shuguang Hospital). A total of 140 participants will be selected from two sites to participate in the study.

With:

The study will be carried out from January 2000 to December 2014, comprised of a 36 month treatment phase and followed by a 10-year extension phase without treatment. Subjects will be randomly assigned to receive BSYJF (n=70) or placebo (n=70) with a 1:1 allocation ratio. The study will carry out at the two sites (Institute of Orthopaedics and Traumatology, Shanghai Academy of TCM; and Shuguang Hospital). **At the beginning of the study, a total of 140 participants will be selected from two sites to participate in the study.**

Section: 6.2. Exclusion Criteria

Add:

For the extension phase, additional exclusion criteria have been defined to protect subjects who have experienced a large bone loss or fragility fracture during the initial 36 month treatment phase of the study.

Section: 9. Drug Administration

Add:

All the included participants will be asked to record every number of fall during 10 years extension follow-up.

Section: 15.2 Secondary Endpoints

Add:

Additional endpoint:

Number of falls at the end of 10 years extension follow-up will be performed by chi-square test.

Section: 17.2 Diet and Exercise Control

Add:

During the entire trial, the subjects will be advised to follow the guideline for daily dietary intake and physical activities, such as intaking high-protein diet, avoiding alcohol abuse and smoking, and keeping active in daily physical activities. The questionnaire for daily dietary intake and physical activities should be recorded at 12, 24, 36 months during 36-month clinical trial **and at the 3rd, 6th and 10th year during 10-year follow-up.** Calcium and vitamin D supplement on their own should be prohibited in 36-month clinical trial.

Section: 12. Table 1 Schedule of Assessments (updated)

Assessment	Screening	Baseline	Treatment			Follow-up		
	Wk. −2-0	Wk.0	Mo. 12	Mo. 24	Mo. 36	Yr. 3rd	Yr. 6th	Yr. 10th
**General information**								
Informed consent	√							
Medical/Medication history	√							
Physical exam (Height & Weight)	√		√	√	√			
BMD screening	√							
**Observations**								
Falls		√	√	√	√	√	√	√
BMD and lean mass		√	√	√	√			
TUG test		√	√	√	√			
Bone biomarkers		√	√	√	√			
Hematology		√	√	√	√			
ECG		√	√	√	√			
B-ultrasound		√	√	√	√			
DDIPA		√	√	√	√	√	√	√
Adverse events			√	√	√			
**Others**								
Drug dispensing		√	√	√				
Drug return			√	√	√			
Combined disease and Medications	√		√	√	√			

25. SUMMARY OF AMENDMENTS

Rationale and Changes (Original Protocol 1.0 to Version 1.1):

The primary endpoint of the number of falls reflects the factorial structure of the current design. The description of the primary analysis approach was updated to be aligned with the previously simple size calculation, and statistical analysis plan.

Rationale and Changes (Original Protocol 1.1 to Version 1.2):

To assess the latent effects of BSYJF interventions on number of falls in late postmenopausal women, eligible subjects will enter a 10 year extension phase at the end of the initial 36 months treatment phase. Some changes were updated to be aligned with the trial.
